# Identification of Key Enzymes and Genes Modulating L-Ascorbic Acid Metabolism During Fruit Development of *Lycium chinense* by Integrating Metabolome, Transcriptome, and Physiological Analysis

**DOI:** 10.3390/ijms252111394

**Published:** 2024-10-23

**Authors:** Chongxin Yin, Huichun Xie, Guigong Geng, Zuxia Li, Jianxia Ma, Xiaozhuo Wu, Quan-Sheng Qiu, Feng Qiao

**Affiliations:** 1 Key Laboratory of Tibetan Plateau Medicinal Plant and Animal Resources, School of Life Sciences, Qinghai Normal University, Xining 810008, China; yinchongxin0701@163.com (C.Y.); yezino.1@163.com (H.X.); 18909789774@163.com (Z.L.); majianxia0926@163.com (J.M.); xiaozhuo0623@163.com (X.W.); 2 Academy of Plateau Science and Sustainability, Qinghai Normal University, Xining 810008, China; 3 Qinghai South of Qilian Mountain Forest Ecosystem Observation and Research Station, Huzhu 810500, China; genggg-298@163.com; 4 Academy of Agricultural and Forestry Sciences, Qinghai University, Xining 810016, China; 5 MOE Key Laboratory of Cell Activities and Stress Adaptations, School of Life Sciences, Lanzhou University, Lanzhou 730000, China; qiuqsh@lzu.edu.cn

**Keywords:** *Lycium chinense*, fruit developing stage, L-ascorbic acid, metabolomics, transcriptomics, physiology

## Abstract

*Lycium chinense* is acknowledged for its substantial nutritional benefits, particularly attributed to the high levels of ascorbic acid (AsA) found in its fruits. The “Mengqi No.1” variety of *L. chinense*, which is cultivated in Qinghai, is known for its high yield and exceptional quality. We utilized the “Mengqi No.1” variety as experimental materials and combined metabolomic, transcriptomic, and physiological analyses to investigate the metabolites, genes, and enzymes related to AsA metabolism in *L. chinense* fruits. The results revealed nine differential metabolites associated with AsA metabolism in *L. chinense* fruits across three stages, including 1D-Myo-Inositol-1,4-Bisphosphate, D-Fructose, L-(+)-Arabinose, I-Inositol, L-Arabinitol, D-Galactose-1-P, lactose, α-D-Glucose, and D-Glucose-6-P. Notably, the contents of D-Glucose-6-P, D-Galactose-1-P, and D-Fructose were increased as the fruit developed. Additionally, fresh weight, longitudinal length, and radial width were increased, while the contents of AsA and DHA were decreased. GalDH and DHAR are critical enzymes for the accumulation of AsA and DHA, exhibiting positive correlation coefficient. Furthermore, *PMM1*, *PMM5*, *GME2*, and *GME3* were identified as key regulatory genes in the L-Galactose pathway of AsA synthesis, influencing D-Galactose-1-P, D-Glucose-6-P, α-D-Glucose, and D-Fructose. *DHAR1* and *DHAR2* are considered key positive regulator genes of AsA and DHA in the AsA-GSH cycle. However, the majority of genes (nine) act as negative regulators of AsA and DHA. These findings provide a foundation for the understanding of the regulatory mechanism of AsA metabolism in *L. chinense* fruits and offer insights into the utilization of AsA from *L. chinense*.

## 1. Introduction

Ascorbic Acid (AsA) is a vital antioxidant molecule that plays a critical role in the metabolic processes of both animals and plants [1,2]. Notably, humans and certain other animals have lost the capacity of AsA biosynthesis and therefore require a dietary source of this essential nutrient [3,4]. Fruits from specific species, such as *Myrciaria dubia* [5], *Malpighia emarginata* [6], *Diospyros kaki* [7], and *Ziziphus jujuba* [8], are particularly rich in AsA. Dynamic changes in AsA content were observed at six different stages of fruit development and ripening in *Actinidia latifolia*, with the AsA content reaching 1108.76 ± 35.26 mg/100 g FW at full maturity [9]. Obtaining AsA from these dietary sources is essential, as it significantly contributes to overall health [10,11,12].

Once introduced into the body, AsA neutralizes reactive oxygen species (ROS) through both enzymatic and non-enzymatic mechanisms [13,14,15]. The biosynthetic route for AsA in plants encompasses several synthesis pathways, including D-Mannose/L-Galactose, D-Galacturonic acid, L-Gulose, and Myo-inositol [16,17,18]. It has been reported that two AsA synthesis pathways—specifically, the L-Galactose pathway and the Myo-inositol pathway—are present in jujube [8]. In kiwifruit, three synthetic pathways have been identified: the L-Galactose pathway, the D-Galacturonate pathway, and the Myo-inositol pathway. Similarly, in *D. kaki* fruits, three pathways have also been discovered [7].

*Lycium chinense*, or goji berry/wolfberry, a member of the Solanaceae family, is a perennial deciduous shrub widely distributed across temperate and subtropical regions [16,19]. Pharmacological research indicates that *L. chinense* may enhance vision, boost immunity, mitigate liver injury, lower blood sugar and lipid levels, reduce cancer risk, provide neuroprotection, and exhibit antioxidant and anti-aging properties [20,21,22,23,24]. It has been reported that goji berry is often used in conjunction with fruits such as jujube, black sesame, and walnuts to create gelatinous cakes, which demonstrate significant effects on mental health, blood deficiency treatments, and conditions related to anemia [25,26].

Metabolome analyses indicated that amino acids, vitamins, and flavonoids exhibited similar accumulation patterns across various developmental stages of fruits. *L. ruthenicum* demonstrated a higher accumulation of metabolites compared to *L. chinense* at the same developmental stage, including L-Glutamate, L-Proline, L-Serine, abscisic acid (ABA), sucrose, thiamine, naringenin, and quercetin [27]. Furthermore, *L. barbarum* contains elevated levels of total phenolics and flavonoids in comparison to *L. ruthenicum*, contributing to its greater antioxidant activity [28]. Notably, *L. barbarum* also exhibited superior antioxidant activity relative to *L. ruthenicum* and possessed higher concentrations of vitamin E and carotenoids [28]. The number of differentially regulated metabolites in pairwise comparisons of developmental stages in *L. chinense* ranged from 66 (stages 3 vs. 4) to 129 (stages 2 vs. 5) [29]. The largest number of differentially regulated metabolites in successive stage comparisons was observed between the 1st and 2nd stages, with a total of 117 metabolites identified [29]. Pectin may constitute the major component of polysaccharides in goji berries, with its content increasing to 235.8 mg/g dry weight (DW) [30]. Proteomic and transcriptomic analyses revealed 6410 differentially expressed genes (DEGs) and 2052 differentially expressed proteins (DEPs), with a notable overrepresentation of gene ontology (GO) terms and KEGG pathways related to flavonoids and polysaccharides [30]. The accumulation of sugars in *L. barbarum* fruits harvested in September was found to be superior, whereas the content of vitamin C and carotenoids was richer in fruits harvested in June or July [31]. The highest AsA content was recorded in July, reaching 74.94 mg/100 g fresh weight (FW), while the lowest content was observed in September [31]. Additionally, the accumulation and expression of AsA, glutamic acid, glutamine, and related enzyme genes varied in wolfberry fruits across different harvesting periods [31]. Both values were significantly higher than the 48.94 mg/100 g FW reported for goji berries [32]. However, there is currently limited research on the regulatory mechanisms of ascorbic acid metabolism in *L. ruthenicum* and *L. chinense*.

In recent years, there has been considerable attention on enhancing the nutritional quality of fruits. Therefore, it is essential to systematically understand the regulatory mechanisms of AsA in *L. chinense* fruits. This study integrates metabolomics, transcriptome data, and the activity of AsA-GSH (glutathione) cycle enzymes, along with various physiological indicators, to detect changes in genes and metabolites during the development of *L. chinense* fruits. We aim to explore the relationships among the key genes involved in AsA metabolism and to elucidate the connections among the enzymes, transcriptome, and metabolome involved in AsA metabolism. This study will enhance our understanding of the biosynthetic mechanisms of AsA and provide valuable insights into genes and metabolites in *L. chinense* fruits.

## 2. Results

### 2.1. Phenotypic Analysis of L. chinense Fruits Across Three Developmental Stages

**Figure 1:**
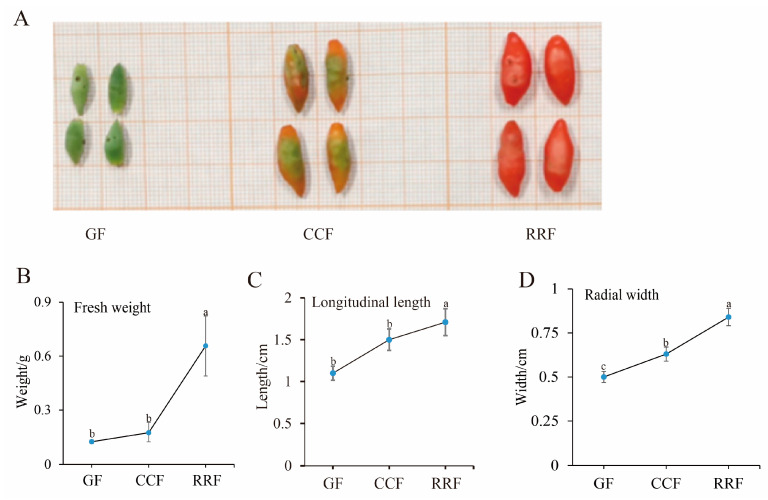
The fruit phenotypic indicators of *L. chinense* were examined at three stages. (**A**) GF (green fruits, 16–19 days after flowering), CCF (change-color fruits, 22–25 days after flowering), and RRF (red-ripe fruits, 31–34 days after flowering). (**B**) Analysis of fresh weight. (**C**) Analysis of longitudinal length. (**D**) Analysis of radial width. Fresh weight, longitudinal length, and radial width were calculated as the mean of ten replicates. Error bars indicating ± standard error (SE) from the replicates. The letters denoted significance levels at *p* ≤ 0.05, as determined by Turkey’s test.

Metabolites were extracted from *L. chinense* fruits at three developmental stages: GF (green fruit, 16–19 days after flowering), CCF (change-color fruit, 22–25 days after flowering), and RRF (red-ripe fruit, 31–34 days after flowering) (Figure 1A). Throughout the development of *L. chinense* fruits, fresh weight, longitudinal length, and radial width exhibited an upward trend; these indicators were increased in RRF compared to GF by 2.65 times, 28%, 34%, respectively (*p* < 0.05, Figure 1B).

### 2.2. Analysis of Metabolites in L. chinense Fruits Across Three Developmental Stages

**Figure 2:**
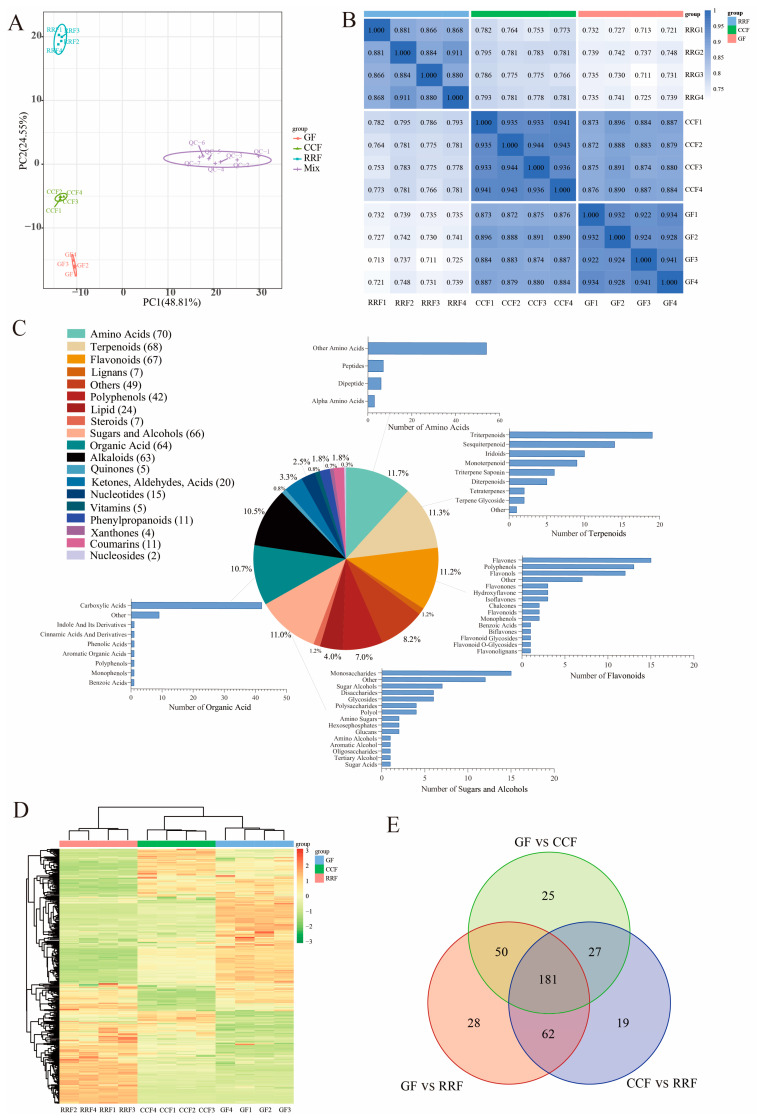
Metabonomic analysis of *L. chinense* fruits at three developmental stages: GF, CCF, and RRF. (**A**) The PCA of *L. chinense* fruits across these three stages was shown. (**B**) A correlation analysis on the metabolites in *L. chinense* fruits across the three stages. (**C**) The types and proportions of the annotated metabolites in *L. chinense* fruits. (**D**) The identified metabolites exhibited distinct accumulation patterns at each stage. The colors represented the accumulation levels of each metabolite, ranging from low (green) to high (red). (**E**) Petal diagrams of DAMs analyzed using OmicShare tools (https://www.omicshare.com/, accessed on 20 August 2024).

Principal Component Analysis (PCA) was employed to assess variability among the 12 samples, including four replicate samples collected from each stage. The findings revealed a close proximity among the four replicate samples within the same stage, while significant differences in metabolite distribution were observed between fruits at different stages (Figure 2A). Overall, the results indicated that RRF samples differed from GF and CCF samples along PC2 (Figure 2A), while GF samples differed from RRF and CCF along both PC2 and PC1. Additionally, correlation analysis indicated a higher degree of correlation within samples compared to that between different samples, thereby confirming the stability and reliability of the metabolome data obtained (Figure 2B).

To further assess the variations in metabolites among the fruits of *L. chinense* across three developmental stages, we employed the criteria of fold change (FC) > 1, *p* < 0.05, and VIP > 1 to identify differentially accumulated metabolites (DAMs). A total of 600 metabolites were detected, which included 70 amino acids, 68 terpenoids, 67 flavonoids, 66 sugars and alcohols, 64 organic acids, 63 alkaloids, 42 polyphenols, 24 lipids, 20 compounds (including ketones, aldehydes, and acids), 15 nucleotides, 11 phenylpropanoids, 11 coumarins, 7 lignins, 7 steroids, 5 vitamins, 5 quinones, 4 oxanthones, 2 nucleosides, and 49 other metabolites (Figure 2C). Among the top five metabolite categories, the proportions of amino acids, terpenes, flavonoids, sugars and alcohols, and organic acids were 11.7%, 11.3%, 11.2%, 11.0%, and 10.7%, respectively (Figure 2C). In the clustering heatmap, the accumulation of 600 metabolites exhibited distinct variations in the patterns of metabolite abundance across different fruit samples (Figure 2D). Notably, among the differentially abundant metabolites, 181 were common across all three groups (Figure 2E). The unique differentially annotated metabolites identified included 25 for GF vs. CCF, 28 for GF vs. RRF, and 19 for CCF vs. RRF (Figure 2E).

A total of 386 metabolites from *L. chinense* fruits successfully matched with the KEGG database during the three developmental stages (Table S1). Specifically, seven metabolites related to fructose and mannose metabolism, eleven associated with galactose metabolism, and five linked to ascorbate and aldarate metabolism were identified, all of which were involved in the AsA synthesis pathway (Table S1). Comparisons revealed 121 up-regulated and 190 down-regulated metabolites between GF and RRF stages, 128 up-regulated and 161 down-regulated metabolites between the CCF and RRF stages, and 112 up-regulated and 171 down-regulated metabolites between GF and CCF stages (Table S2).

### 2.3. Nine Differential Metabolites in the AsA Synthesis Pathway of L. chinense Fruits Across Three Stages

**Figure 3:**
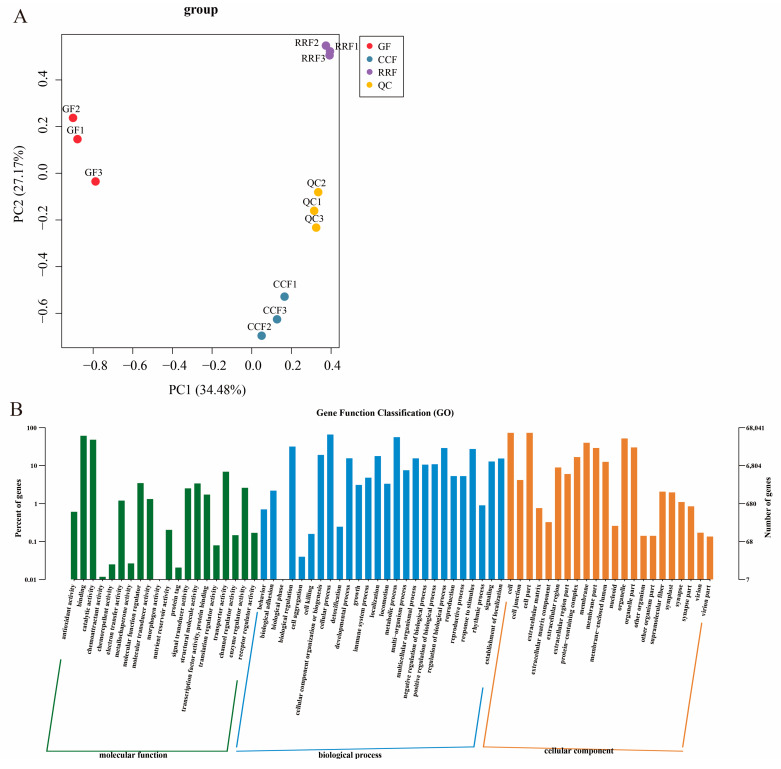
Transcript analysis of *L. chinense* fruits at three developmental stages: GF, CCF, and RRF. (**A**) The PCA of *L. chinense* fruits across these three stages was shown. (**B**) Gene function classification (GO) of *L. chinense* fruits.

In the analysis of DAMs in *L. chinense* fruits at three developmental stages, a total of 66 metabolites were identified, including 15 monosaccharides, 7 sugar alcohols, 6 disaccharides, 6 glycosides, 4 polyols, 4 polysaccharides, 2 glucans, 2 sugar acids, 2 hexose phosphates, 2 amino sugars, 1 amino alcohol, 1 tertiary alcohol, 1 oligosaccharide, 1 aromatic alcohol, and 12 others (Class II, Table S3). Among these, nine differential metabolites related to AsA metabolism were identified: 1D-Myo-Inositol-1,4-Bisphosphate (1D-Myo-Inositol-1,4-BP), D-Fructose, L-(+)-Arabinose, I-Inositol, L-Arabinitol, D-Galactose-1-Phosphate (D-Galactose-1-P), lactose, α-D-Glucose, and D-Glucose 6-Phosphate (D-Glucose-6-P) (Table 1). The ratio of D-Fructose content in RRF/GF and CCF/GF was significant, measuring 12.38 and 7.66, respectively. In contrast, the lactose content in RRF/GF and CCF/GF was relatively low, recorded at 0.05 and 1.40, respectively (Table 1). The heatmap presented nine DAMs related to AsA metabolism was shown in Figure 3A. The results indicated:

- In GF, one metabolite (lactose) exhibited a high concentration, while two metabolites (L-(+)-Arabinose, and D-Fructose) were presented at low concentrations.
- In CCF, three metabolites (L-(+)-Arabinose, I-Inositol, and lactose) showed high concentrations, whereas two metabolites (α-D-Glucose, and 1D-Myo-Inositol-1,4-BP) were found at low concentrations.
- In RRF, five metabolites (lactose, 1D-Myo-Inositol-1,4-BP, D-Glucose-6-P, D-Galactose-1-P, and D-Fructose) exhibited high concentrations.

These results indicated a greater abundance of differential metabolites associated with the AsA synthesis pathway in the later stage of RRF in *L. chinense* fruits.

### 2.4. Analysis of the Transcripts of AsA Metabolism of L. chinense Fruits

Transcriptome sequencing was conducted for three developmental stages of *L. chinense* fruits, with three replicates for each stage, yielding a total of nine samples (Table S4). The sequencing was generated between 42,916,958 and 48,531,116 raw reads. The sequences with Q30 (base accuracy of 99.9%) constituted over 95.41% of the total, while those with Q20 (base accuracy of 99.0%) exceeded 98.79%. Additionally, the GC content was greater than 43.25% (Table S4). Transcript analysis of *L. chinense* fruits at three developmental stages is shown in Figure 3. PCA was employed to assess variability among 9 samples, with three replicates per group (Figure 3A). The findings revealed a close proximity between the three replicate samples within the same stage, while significant differences in transcript distribution were observed between different-stage fruits (Figure 3A). The results indicated that GF, CCF, and RRF samples differed along PC1 and PC2 (Figure 3A). Gene function classification (GO) of *L. chinense* fruits was displayed in Figure 3B. The molecular function was focused on binding, catalytic activity, and transporter activity; the biological process focused on cellular process, metabolic process, and biological regulation; and the cellular component focused on cells, cell parts, and organelles (Figure 3B).

**Figure 4:**
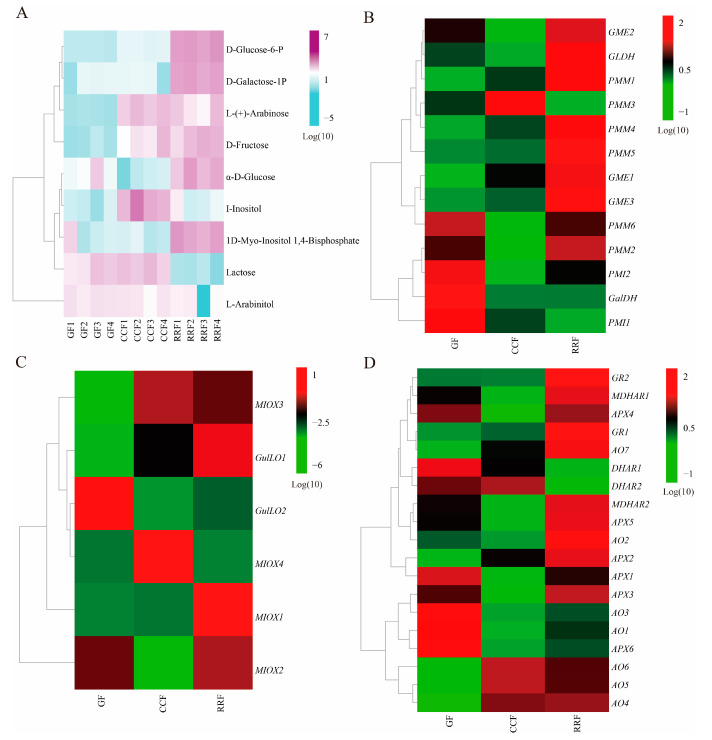
The heatmap and hierarchical cluster illustrating the DAMs and DEGs associated with AsA synthesis pathway in *L. chinense* fruits across three developmental stages. (**A**) The heatmap of the DAMs related to AsA metabolism, with colors representing the accumulation levels of each metabolite, ranging from low (light green) to high (purple). (**B**) The heatmap of the DEGs involved in the L-Galactose pathway of AsA synthesis. (**C**) The heatmap of the DEGs associated with the L-Gulose and Myo-inositol pathways of AsA synthesis. (**D**) The heatmap of the DEGs related to the AsA-GSH cycle, which is crucial for the oxidation and reduction of AsA. Colors indicate the accumulation levels of each transcript, from low (green) to high (red).

Previous studies have demonstrated that AsA synthesis originates from the D-Galacturonate pathway, L-Galactose pathway, L-Gulose pathway, and Myo-inositol pathway [33,34,35]. These pathways yield early precursors, specifically L-Galactono-1,4-lactone and L-Gulose-1,4-lactone compounds. Subsequently, the oxidation and reduction of AsA are involved in the final AsA-GSH cycle. In this study, we reconstructed the AsA metabolism and identified the abundance of thirty-eight transcripts encoding twelve key enzyme-coding genes associated with AsA metabolism (Table S5). As shown in Figure 4B, ten key enzymes were coded by more than one unigene: *PMI* (2), *PMM* (6), *GME* (3), *GulLO* (2), *MIOX* (4), *APX* (6), *AO* (7), *MDHAR* (2), *DHAR* (2), and *GR* (2) (Table S5). Two key enzymes were coded by a single unigene: *GaLDH* (1) and *GLDH* (1) (Table S5). Cluster analysis of all differentially expressed genes involved in AsA synthesis and cycle pathways across different developmental stages revealed considerable variability in gene expression as the fruit developed (Figure 4B–D, Table S6). In the L-Galactose synthesis pathway, transcript levels of *PMI2*, *GalDH*, and *PMI1* were high in GF, whereas *PMM1*, *PMM4*, and *GME1* exhibited low expression (Figure 4B). The expression of *PMM3* was high in CCF, while *PMI2*, *GME2*, *GLDH*, *PMM6*, *PMM2*, and *PMI2* showed low levels (Figure 4B). In RRF, *GLDH*, *PMM1*, *PMM4*, *PMM5*, and *GME3* were highly expressed, while *PMM3* and *PMI1* were low (Figure 4B). In the L-Gulose and Myo-inositol synthesis pathways, *GulLO2* was highly expressed, while *MIOX3* and *GulLO1* were low in GF (Figure 4C). *MIOX4* expression was high, whereas *GulLO2* and *MIOX2* were low in CCF (Figure 4C). In RRF, *GulLO1* and *MIOX1* were high, while *MIOX4* was low (Figure 4C). Within the AsA-GSH cycle, *DHAR1*, *AO3*, *AO1*, and *APX6* were high in GF, while *AO7*, *AO6*, *AO5*, and *AO4* were low (Figure 4D). Additionally, *MDHAR1*, *APX4*, *MDHAR2*, *APX5*, *APX1*, *APX3*, and *AO1* were low in CCF (Figure 4D). *GR2*, *GR1*, and *AO2* were high in RRF, while *DHAR1* and *DHAR2* were low (Figure 4D). Among the 38 genes analyzed, transcript expression of 19 genes was high in RRF, 9 in GF, and 2 in CCF (Figure 4B–D). These results indicated that half of the genes in the AsA pathway were active during the late-stage RRF of *L. chinense* fruits.

### 2.5. Validation of the Differentially Expressed Genes in AsA Metabolism of L. chinense Fruits by RT–qPCR

A total of 23 genes of the 38 differentially expressed genes in the AsA metabolic pathway were selected for validation via RT-qPCR to confirm the reliability and accuracy of the RNA-seq data (Table S7). The *GAPDH* housekeeping gene was utilized as an endogenous reference for normalizing the expression levels of the target genes. The results indicated that the relative expressions of all selected genes measured by RT-qPCR were consistent with the transcript expressions obtained from the RNA-seq data, thereby reinforcing the authenticity and reliability of the transcriptome data.

**Figure 5:**
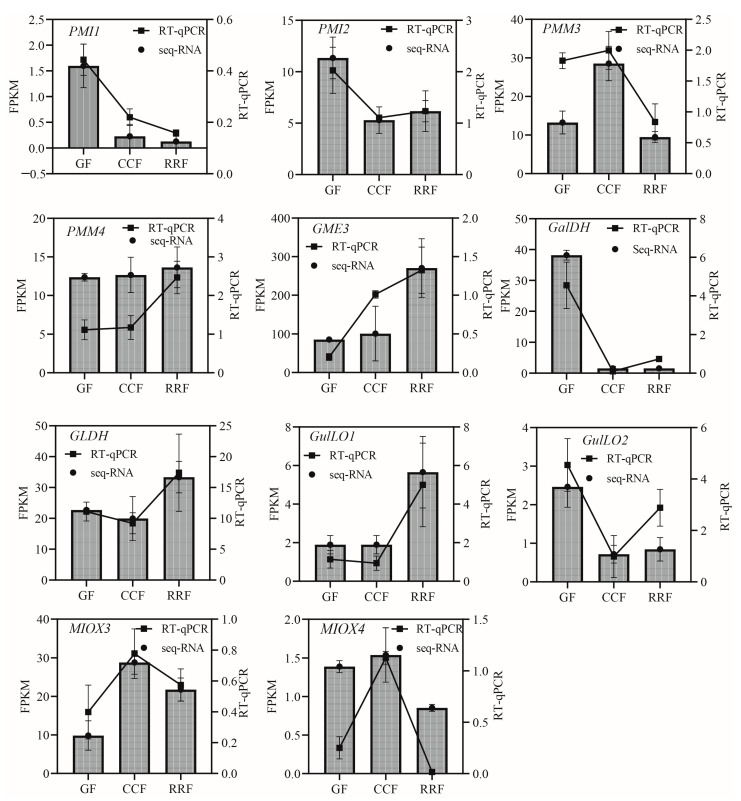
The expression of genes in the L-Galactose, L-Gulose, and Inositol pathway, which are parts of the ASA synthetic pathway, were analyzed by RT-qPCR in *L. chinense* fruits at three developmental stages: GF, CCF, and RRF. The column chart displayed the FPKM values for each gene, while the line chart illustrated the relative gene expression by RT-qPCR. Relative gene expression was calculated using the 2^−ΔΔct^ method. Vertical bars represented means ± SD from three replicates.

**Figure 6:**
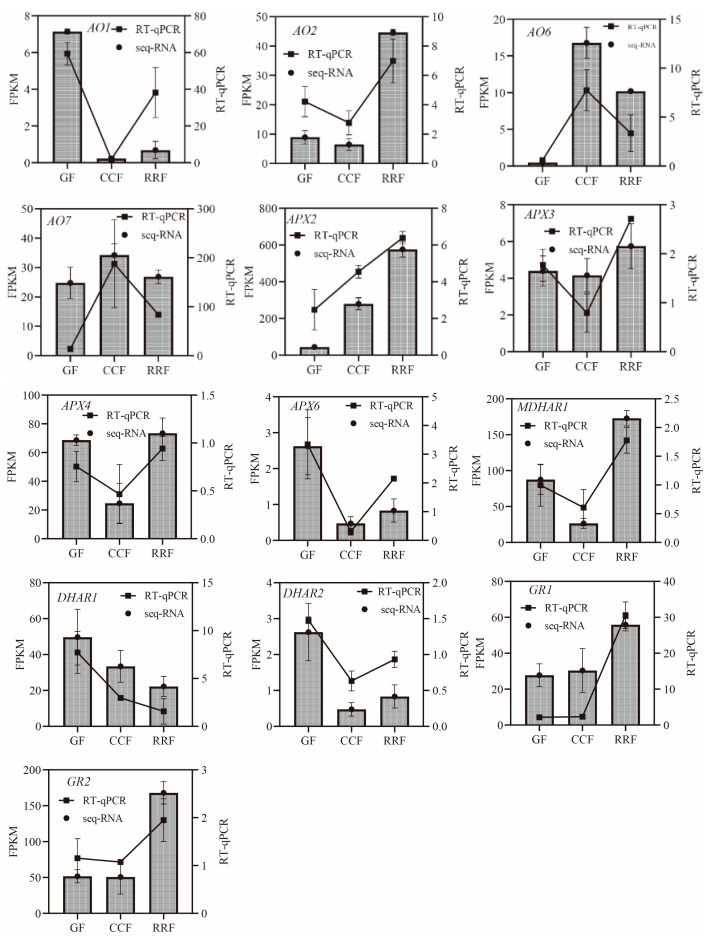
The expression of genes in the ASA-GSH cycle, which is part of the AsA oxidation and reduction pathway, was analyzed by RT-qPCR in *L. chinense* fruits at three developmental stages: GF, CCF, and RRF. The column chart illustrated the FPKM values of the genes, while the line chart depicted the relative gene expression by RT-qPCR. Relative gene expression was calculated using the 2^−ΔΔct^ method. Vertical bars represented the means ± SD from three replicates.

In the L-Galactose synthesis pathway, the relative expressions of *PMI1*, *PMI2*, and *GalDH*, as assessed by RT-qPCR, exhibited a decreasing trend, whereas those of *PMM4*, *GME3*, and *GLDH* showed an increasing trend (Figure 5). Notably, *PMM3* displayed an initial rise followed by a decline (Figure 5). In the L-Gulose and Myo-inositol pathways, *GulLO1* demonstrated an increasing trend (Figure 5). *MIOX3* and *MIOX4* exhibited an initial increase followed by a decrease, while *GulLO2* showed an initial decrease followed by an increase (Figure 5). In the AsA-GSH cycle, *APX2*, *GR1*, and *GR2* displayed an increasing trend (Figure 6). *AO6* and *AO7* initially increased before decreasing, whereas *AO1*, *AO2*, *APX3*, *APX4*, *APX6*, *DHAR2*, and *MDHAR1* exhibited a trend of initial decrease followed by an increase (Figure 6).

### 2.6. Analysis of Enzyme Activities Involved in the AsA-GSH Cycle of L. chinense Fruits Across Three Stages

**Figure 7:**
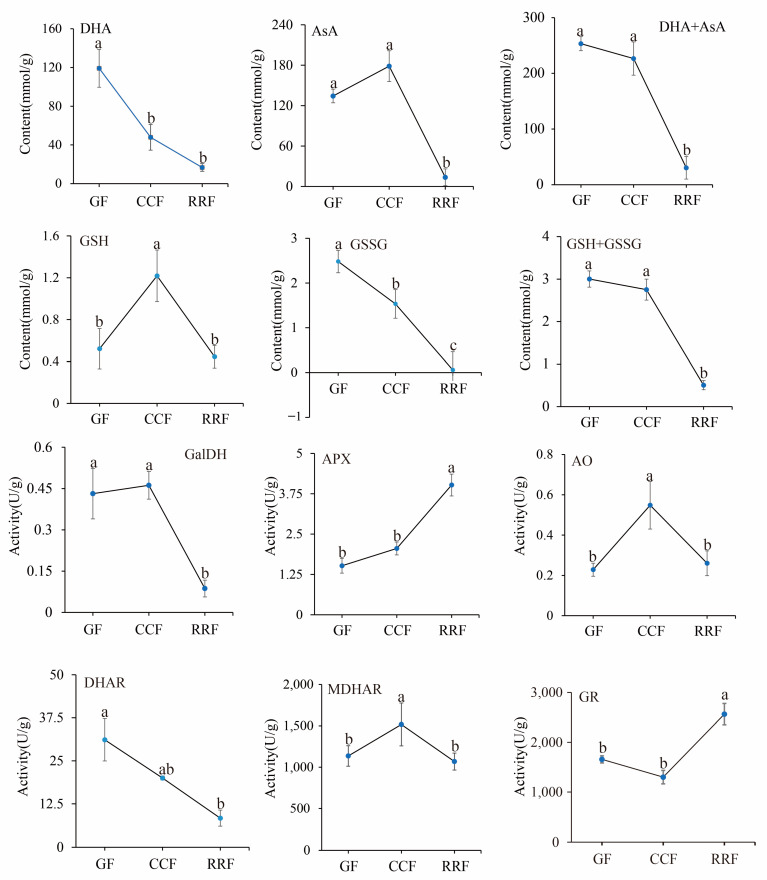
The analysis of enzyme activity of the AsA-GSH cycle and AsA content was conducted with *L. chinense* fruits at three developmental stages: GF, CCF, and RRF. Each value reported was the mean of three replicates, with error bars indicating ± SE from these replicates. The letters denoted significance levels at *p* ≤ 0.05, as determined by Turkey’s test.

12 physiological indices were analyzed in *L. chinense* fruits. The contents of four compounds (AsA, DHA, GSSG, and GSH) and the activities of six enzymes (GalDH, APX, AO, DHAR, MDHAR, and GR) were determined using an enzyme-linked immunosorbent assay (ELISA). The activities of these six enzymes in the AsA-GSH cycle were assessed in *L. chinense* fruits at three developmental stages (Figure 7). The contents of DHA, AsA+DHA, and GSSG displayed a decreasing trend, with the highest values recorded at 119.12 mmol/g, 253.37 mmol/g, and 2.48 μmol/g in GF, respectively (Figure 7). The content of GSH initially increased and then declined, peaking at 1.22 μmol/g in CCF (Figure 7).

The activities of the APX and GR enzymes demonstrated an increasing trend, reaching maximum activities of 4.02 U/g and 2564.76 U/g in RRF, respectively (Figure 7, *p* < 0.05). Conversely, the activities of GalDH and DHAR exhibited a decreasing pattern, with high activities of 0.46 U/g in CCF and 31.13 U/g in GF, respectively. The activities of GalDH and DHAR in RRF were decreased by 79% and 73% compared with GF, respectively (Figure 7, *p* < 0.05). The activities of AO and MDHAR showed an initial increase followed by a decline. The highest activity of AO was 0.55 U/g in CCF, and the highest activity of MDHAR was 1517.12 U/g in CCF (Figure 7, *p* < 0.05).

### 2.7. Correlation Analysis Between Physiological Indices and Gene Expression Related to the AsA-GSH Cycle in L. chinense Fruits

**Figure 8:**
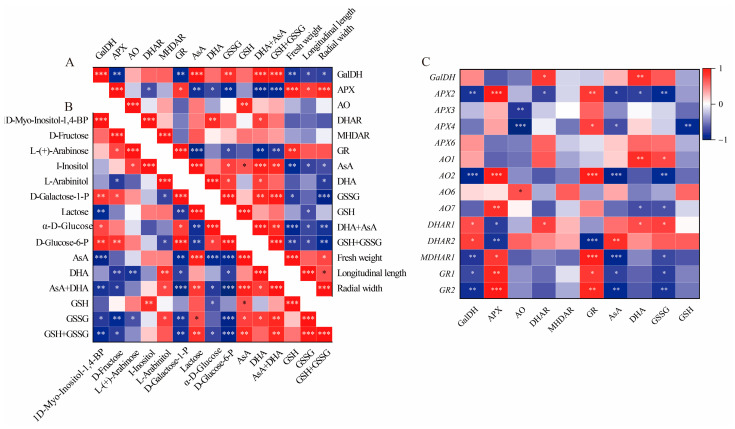
Correlation analysis between physiological indices and gene expression involved in the AsA-GSH cycle of *L. chinense* fruits. (**A**) Heatmap of fifteen physiological indicators. (**B**) Correlation of fifteen metabolites involved in AsA metabolism. (**C**) Correlation of six enzymes, AsA, GSSG and gene expressions. Red and blue colors denoted positive and negative correlations, respectively. This correlation method was calculated using the Origin Correlation Plot plugin. The significance levels were indicated as follows: *: 0.01 < *p* < 0.05; **: 0.001 < *p* < 0.01; ***: *p* ≤ 0.001.

In this study, the total AsA content, which includes both AsA and DHA, was accumulated in GF and CCF, but was decreased in RRF. This trend aligns with the accumulation patterns observed in *Z. jujuba*, kiwifruit, and peach fruits [8,36,37]. The AsA content was assessed at three different stages of fruit development and ripening in *L. chinense*, with total AsA levels reaching 253.38 mmol/g (456.08 mg/100g fresh weight, FW) in GF, 226.59 mmol/g (407.84 mg/100g FW) in CCF, and 30.37 mmol/g (54.65 mg/100g FW) in RRF. In the ripe stage, the total AsA content of 54.65 mg/100g FW observed in our study was higher than the 48.94 mg/100g FW reported for goji berries by Teixeira [32], but lower than the 74.94 mg/100g FW found in wolfberry fruits reported by Liu [31]. In our study, the total AsA and total GSH, comprising both oxidized (GSSG) and reduced forms, were positively correlated with the activities of GalDH (*p* < 0.01), DHAR (*p* < 0.05), the contents of GSSG (*p* < 0.01), and AsA (*p* < 0.001). Conversely, the total AsA and total GSH exhibited an inverse correlation with the activities of APX (*p* < 0.001) and GR (*p* < 0.01) (Figure 8A). The activities of APX and GR were positively correlated with the expression of *APX2* (*p* < 0.001, 0.01), *AO2* (*p* < 0.001), *MDHAR1* (*p* < 0.05, 0.001), *GR1* (*p* < 0.01, 0.05), and *GR2* (*p* < 0.001, 0.05), but inversely correlated with the expression of *DHAR2* (*p* < 0.001, 0.05) (Figure 8C). Additionally, GalDH activity was positively correlated with the expression of *DHAR2* (*p* < 0.05), while it was inversely correlated with the expressions of *APX2* (*p* < 0.01), *AO2* (*p* < 0.001), *MDHAR1* (*p* < 0.01), *GR1* (*p* < 0.05), and *GR2* (*p* < 0.01) (Figure 8C). The activity of APX was consistently high in relation to the expression of the gene *APX3* (*p* < 0.001), while AO activity was associated with *AO6* expression (*p* < 0.05), and GR activity corresponded with the expressions of *GR1* (*p* < 0.05) and *GR2* (*p* < 0.01) (Figure 8C). Therefore, GalDH and DHAR can be regarded as important enzymes in the accumulation of high levels of ascorbate in *L. chinense* fruits, whereas APX and GR may play significant roles in inhibiting AsA accumulation.

At the mature green stage of pepper fruits, AsA was highly positively correlated with D-galacturonic acid (*p* < 0.01) but inversely correlated with L-ornithine (*p* < 0.01) and DHA (*p* < 0.05) [38]. In the mature red stage, AsA was positively correlated with I-inositol, D-Glucose, D-Galacturonic acid, D-Glucuronic acid, and L-Gulono-1,4-lactone (*p* < 0.05), but negatively correlated with L-Glutamic acid (*p* < 0.05) [38]. In our study, the total AsA (AsA+DHA) was positively correlated with lactose (*p* < 0.01), L-Arabinitol (*p* < 0.05), AsA (*p* < 0.001), and DHA (*p* < 0.05), but inversely correlated with 1D-Myo-Inositol-1,4-BP (*p* < 0.01), D-Fructose (*p* < 0.05), D-Galactose-1-P (*p* < 0.001), α-D-Glucose (*p* < 0.05), and D-Glucose-6-P (*p* < 0.001) (Figure 8B). Therefore, lactose, L-Arabinitol, AsA, and DHA can be considered important factors in the accumulation of high levels of ascorbate in *L. chinense* fruits.

### 2.8. Correlation Analysis Between the Expressions of 38 Unigenes and 15 Metabolites Involved in AsA Metabolism in L. chinense Fruits

**Figure 9:**
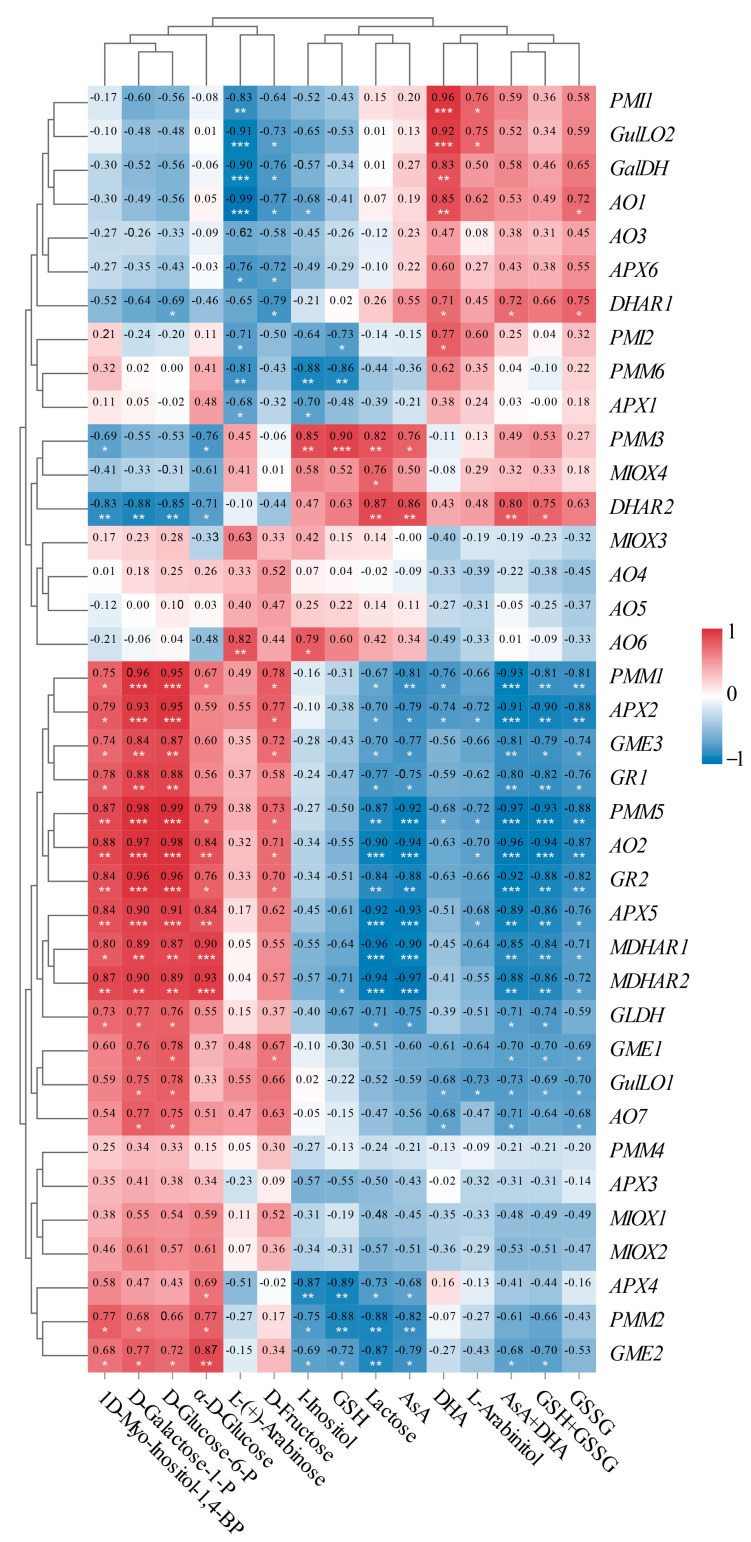
Correlation analysis between the expressions of 38 unigenes and 15 metabolites involved in AsA metabolism in *L. chinense* fruits. Red and blue colors indicated positive and negative correlation, respectively. The significance levels were denoted as follows: * indicated 0.01 < *p* < 0.05; ** indicated 0.001 < *p* < 0.01; and *** indicated *p* ≤ 0.001.

To enhance our understanding of the molecular mechanisms underlying AsA accumulation in *L. chinense* fruits at various developmental stages, we analyzed the pathway relationship between differential metabolites and genes involved in the AsA pathway using transcriptome and metabolome data (Figure 9). This analysis focused on the association between 38 relevant genes and 15 compounds related to AsA in *L. chinense* fruits (Figure 9). Considering the molecular regulation of AsA accumulation in *L. chinense* fruits across three stages, we statistically analyzed five genes (*PMI*, *PMM*, *GME*, *GalDH*, and *GLDH*) involved in the L-Galactose pathway, one gene (*GulLO*) in the L-Gulose pathway, one gene (*MIOX*) in the Myo-inositol pathway, and five genes (*APX*, *AO*, *MDHAR*, *DHAR*, and *GR*) in the AsA-GSH cycle from the three stages, GF, CCF, and RRF (Figure 9). As illustrated in Figure 9, a significant correlation was observed between AsA-related compounds and differential genes. The genes *PMM1*, *PMM5*, *AO2*, *GR2*, *APX5*, *MDHAR2*, and *GME2* exhibited significantly positive correlations with 1D-Myo-Inositol-1,4-BP, D-Glucose-6-P, D-Galactose-1-P, and α-D-Glucose, while these genes displayed significantly negative correlations with lactose, AsA, total AsA, and total GSH (Figure 9). Five genes (*PMI1*, *GulLO2*, *GalDH*, *AO1*, and *PMI2*) showed significantly positive correlations with L-(+)-Arabinose, whereas these genes exhibited significantly negative correlations with DHA (Figure 9). Additionally, two genes (*DHAR1* and *DHAR2*) displayed significantly positive correlations with AsA and total AsA, while nine genes (*APX2*, *APX5*, *GR1*, *GR2*, *AO2*, *AO7*, *GLDH*, *MDHAR1*, and *MDHAR2*) exhibited significantly negative correlations with these compounds (Figure 9).

There was a significantly positive correlation between APX activity and *APX2* expression (*p* < 0.001), DHAR activity and *DHAR1* expression (*p* < 0.05), GR activity and *GR1* (*p* < 0.05), and *GR2* (*p* < 0.01) expressions. However, there was no significant positive correlation between GalDH activity and *GalDH1* expression, or MDHAR activity and *MDHAR* gene expression.

In our study, the gene expression of *PMM1* or *PMM5* displayed (very) significantly positive correlations with four compounds, D-Galactose-1-P, D-Glucose-6-P, D-Fructose, and α—D-Glucose in L-galactose pathway with relative index 0.67–0.99 (*p* < 0.05, *p* < 0.001). *GME2* or *GME3* were significantly positively correlated with three compounds in the L-galactose pathway with relative index 0.72–0.87 (*p* < 0.05, *p* < 0.001). Therefore, *PMM1*, *PMM5*, *GME2*, and *GME3* were identified as key regulatory genes involved in the synthesis of AsA via the L-Galactose pathway. In contrast, the *MIOX1-4* genes did not exhibit key regulatory roles in AsA synthesis through the Myo-inositol pathway. Additionally, in AsA-GSH cycle, *DHAR2* was significantly positively correlated with AsA and AsA+DHA total, while *DHAR1* was significantly positively correlated with DHA and AsA+DHA total, while *APX2*, *AO2*, and *GR2* were identified as major negative regulatory genes affecting AsA regeneration.

## 3. Discussion

Metabolomics has been extensively employed in the study of plant fruits to ascertain the types and concentrations of nutrients present. A total of 388 and 411 metabolites were identified in the pericarp of pepper fruits at the mature green and red stages, respectively [38]. At the mature green stage, fifteen metabolites related to AsA biosynthesis and cycle were detected, while fourteen such metabolites were identified at the red stage [38]. During the mature green stage, nitrogen (N) application led to a decline in AsA synthesis precursors, including D-Glucose and D-Glucose-6-P from the L-Galactose pathway, D-Galacturonic acid from the D-Galacturonate pathway, D-Glucuronic acid from the Myo-inositol pathway, and L-Gulono-1,4-lactone, which is shared between the L-Gulose and Myo-inositol pathways. This decline exhibited a remarkable parallelism with AsA levels [38]. Similar trends were observed at the mature red stage for Myo-inositol and all the aforementioned precursors with the exception of D-Glucose-6-P, which was up-regulated under N250 but down-regulated under N0 and N400 [38]. The initial KEGG analysis of metabolites indicated that L-Galactose metabolism constitutes the primary biosynthetic pathway for AsA in persimmon fruit [7]. Notably, significant differences in the content of L-Galactose pathway-related metabolites were observed in developing PCA fruits, with higher levels of lactose, D-Tagatose, and D-Sorbitol in PCA compared to PCNA [7].

**Figure 10:**
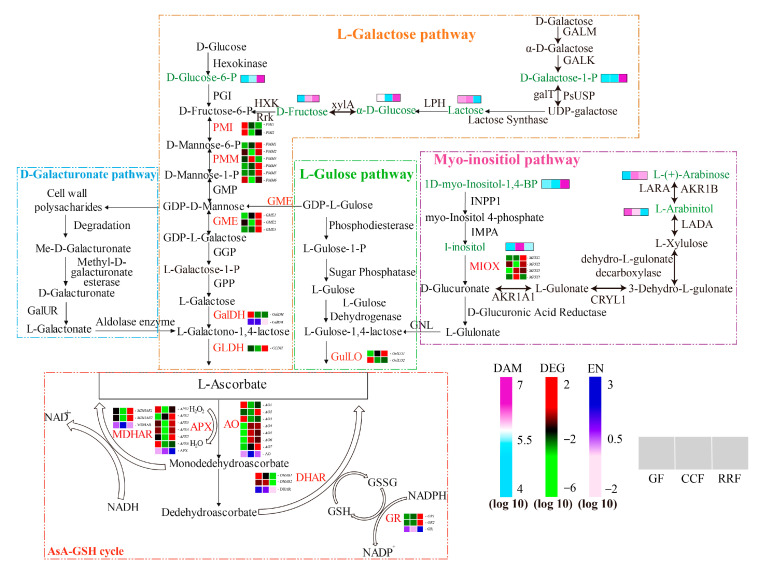
Expression of genes involved in AsA biosynthesis in *L. chinense* fruits across three developmental stages: GF, CCF, and RRF. The AsA biosynthesis pathways examined include the L-Galactose pathway, the D-Galacturonate pathway, the L-Gulose pathway, and the Myo-inositol pathway. Nine green words represented the differential metabolites associated with AsA metabolism, while twelve red words denoted the enzymes involved in this process. The color coding indicated the accumulation levels of DAM, ranging from low (light blue) to high (purple), as well as the accumulation levels of DEG, from low (green) to high (red). The color coding indicated the accumulation levels of EN (enzyme activity), ranging from low (pink) to high (deep blue). The blocks, arranged from left to right, correspond to the GF, CCF, and RRF stages. The heatmap illustrated the normalized FPKM values, standardized by Z-score.

In this study, we identified nine metabolites associated with the AsA synthesis pathway in *L. chinense* fruits (Figure 10). Among these compounds, the levels of D-Glucose-6-P, D-Galactose-1-P, L-(+)-Arabinose, D-Fructose, α-D-Glucose, and 1D-Myo-Inositol-1,4-Bisphosphate were significantly increased in the RRF stage, with increases of 4.10 times, 2.75 times, 1.50 times, 12.38 times, 1.47 times, and 1.51 times compared to those in the GF stage, respectively (Figure 10). The content changes of D-Fructose, D-Galactose-1-P, and D-Glucose-6-P were all more significant than other metabolites (Figure 10). What is more, there were positive correlations among these three metabolites (Figure 8, *p* < 0.05, *p* < 0.01, *p* < 0.001). Simultaneously, AsA content showed negative correlations with D-Galactose-1-P, α-D-Glucose, and D-Glucose-6-P contents (Figure 8, *p* < 0.01, *p* < 0.001). In the mature red stage of pepper fruits, AsA was positively correlated with I-inositol, D-Glucose, D-Galacturonic acid, D-Glucuronic acid, and L-Gulono-1,4-lactone (*p* < 0.05) [38]. We suggested that D-Fructose, D-Galactose-1-P, and D-Glucose-6-P at high levels can make for the AsA precursor synthesis of the L-Galactose pathway in *L. chinense* fruits and do not benefit the AsA-GSH cycle. The different correlation between AsA content and metabolites of AsA precursor synthesis in *L. chinense* and pepper fruits may be due to different species.

The key enzymes involved in AsA biosynthesis are GalDH and GLDH, which are found in various fruits and vegetables [39,40]. Genetic studies have identified critical genes, including *GalDH* and *GLDH*, which play a significant role in AsA biosynthesis, presenting opportunities for crop improvement [39]. In kiwifruit (*Actinidia eriantha*), the activity levels of GalDH, GLDH, MDHAR, and DHAR correlated with the trends in AsA accumulation [41]. It is speculated that GalDH and GLDH are pivotal enzymes in AsA biosynthesis, while MDHAR and DHAR are essential for the AsA regeneration cycle, collectively regulating AsA accumulation in kiwifruit [41]. Furthermore, the high levels of AsA found in wild jujube correlate with an increased abundance of the proteins GalDH, ZjAPXs, and MDHAR1, which are involved in the biosynthesis and recycling pathways of ascorbic acid [42].

The expression patterns observed in leaves and fruits were distinct, further confirming that AsA biosynthesis is differentially regulated across various plant organs [43]. In kiwifruit (*Actinidia eriantha*), 133 unigenes associated with AsA and aldehyde metabolism pathways, along with 23 candidate genes related to AsA biosynthesis, cycling, and degradation, were identified [41]. Within the AsA synthesis and cycling pathways, the genes *PMI*, *PMM*, *GMP*, *GME*, *GGP*, *GPP*, *GalDH*, and *GLDH*, along with their encoded enzymes, have been well characterized, demonstrating their involvement in AsA biosynthesis [44,45]. *GME* has been identified as a key regulatory gene in the synthesis of AsA via the L-Galactose and L-Gulose pathways [46]. Genes *GGP/VTC2*, *GPP*, and *GMP2*, which are associated with the L-Galactose pathway, exhibited a strong correlation with total AsA content, while the genes *MDHAR1*, *APX2*, *APX3*, and *GalDH* showed a positive correlation with total AsA levels in pepper fruits [43]. Gene expansions were detected not only in *PMM*, *GMP*, and *GGP* within the L-Galactose pathway—the primary route for ascorbic acid biosynthesis—but also in genes involved in the recycling of ascorbic acid, such as *AO*, *APX*, and *MDHAR* [9]. Overexpression of the *GGP1* and *GPP* genes resulted in enhanced ascorbate content and improved nutritional quality of tomatoes [47]. In tomatoes, five genes encoding MIOX proteins with MIOX motifs were identified [48]. Furthermore, transgenic lines overexpressing the *MIOX4* gene in tomatoes demonstrated a significant increase in total ascorbate levels in both leaves and red fruits compared to controls [48]. Kiwifruit (*Actinidia eriantha Benth*.) ‘White’ was a novel cultivar known for its elevated levels of L-ascorbic acid (AsA), primarily synthesized through the L-galactose pathway supplemented by the D-galacturonic acid pathway and AsA recycling [49]. The activity of GalDH and the relative expressions of the genes *GMP*, *GPP*, *GalDH*, and *GalUR* were crucial for the regulation of AsA biosynthesis, while the activity and expression of DHAR were primarily responsible for the regulation of AsA recycling in kiwifruit ‘White’ during postharvest [49]. In citrus fruits, the D-galacturonic acid pathway appeared to be relevant in petals, whereas in leaves, the L-galactose pathway (*GGP* and *GME*) also contributed to AsA accumulation [50]. In the flavedo, AsA content was positively correlated with the expression of *GGP* from the L-galactose pathway and negatively correlated with the *DHAR1* gene from the recycling pathway [50]. In the pulp, AsA was primarily regulated by the interplay between the D-galacturonic acid pathway and the *MIOX* and *GalDH* genes [50].

Our results indicated that the synthesis of AsA in goji berry fruit predominantly occurs through the L-galactose pathway, aligning with findings from Alós [50]. In the AsA-GSH cycle, the correlation coefficient between GalDH enzyme activity and AsA content was 0.97 (*p* < 0.001), while the total AsA+DHA was 0.93 (*p* < 0.001). The correlation coefficient between DHAR enzyme activity and DHA content was 0.87 (*p* < 0.01), and with the total AsA+DHA, it was 0.76 (*p* < 0.05). These results suggested that both GalDH and DHAR enzymes played key roles in AsA accumulation, consistent with the findings of Jiang [49]. Furthermore, the expression of the *DHAR* gene was a major regulatory factor in the AsA cycle of goji berry fruits, consistent with Jiang’s research, but contrasting with the findings of Alós [50].

## 4. Materials and Methods

### 4.1. Plant Materials

*L. chinense* variety ‘Mengqi No.1’ was cultivated at Nuomuhong Farm in the Qaidam Basin (36°23′26.84″ N, 94°26′49.04″ E, 2745 m altitude, Qinghai, China). The region is characterized by dry air, prolonged sunshine duration, and significant temperature fluctuations between day and night, with a maximum temperature of 35.8 °C and a minimum of −31 °C. The annual rainfall averages 58.51 mm. The duration from flowering and fertilization to fruit maturity is approximately 28 to 35 days. The fruit development of *L. chinense* can be categorized into three distinct phases (Figure 1): green fruits (GF, 16–19 days after flowering), change-color fruits (CCF, 22–25 days after flowering), and red-ripe fruits (RRF, 31–34 days after flowering). The fruits were uniform in size, fully developed, and free from diseases and pests. All samples were frozen in liquid nitrogen and stored at −80 °C until further analysis.

### 4.2. Preparation of Samples from the Fruits of L. chinense

Samples were extracted from the fruits of *L. chinense* at three distinct stages. First, the samples were subjected to vacuum freeze-drying and weighed to 50 mg, after which 1000 μL of extraction solution (methanol:acetonitrile:water = 2:1:1) was added. Subsequently, the sample was ground using a grinder at 45 Hz for 10 min and sonicated in an ice water bath for an additional 10 min. Afterward, the sample was placed in a −20 °C refrigerator for 1 h. The stationary samples were then transferred to a centrifuge and centrifuged at 4 °C and 12,000× *g* rpm for 10 min. The supernatant (500 μL) was collected and dried using a vacuum concentrator, followed by the addition of 160 μL of a 50% acetonitrile solution to dissolve the dried extract. The dissolved samples were mixed by vortexing, placed again in an ice water bath for 10 min, and centrifuged once more at 4 °C and 12,000× *g* rpm for 10 min. Finally, the supernatant (120 μL) was collected and stored in a 2 mL injection bottle, with 10 μL from each sample combined to create a QC sample for subsequent machine detection.

### 4.3. LC–MS/MS Analysis

LC–MS/MS analysis was conducted using a Whatsch Acquisition I-Class PLUS ultrahigh-performance liquid chromatography system coupled with an AB Sciex Qtrap 6500+ high-sensitivity mass spectrometer. The ultrahigh-performance liquid chromatography conditions were as follows: the chromatographic column utilized was a Waters Acquisition UPLC HSS-T3 (1.8 µm, 2.1 mm × 100 mm). Mobile Phase A comprised ultrapure water containing 0.1% formic acid and 5 mM ammonium acetate, while Mobile Phase B consisted of acetonitrile with 0.1% formic acid added. A gradient elution program was implemented for sample measurement, beginning with initial conditions of 98% A and 2% B, maintained for 1.5 min, followed by an adjustment to 50% A and 50% B over 5 min. The linear gradient was further adjusted to 2% A and 98% B within 9 min, maintained for 1 min, then reverted to 98% A and 2% B within 1 min, and sustained for an additional 3 min. The flow rate was set at 350 μL/min, and the column temperature was maintained at 50 °C. The effluent was alternately directed to an ESI-triple quadrupole-linear ion trap (QTRAP)-MS.

The ESI source operation parameters were as follows: ion source temperature was set to 550 °C; ionizing spray voltage (IS) was 5500 V in positive ion mode and −4500 V in negative ion mode. The ionization source gas I (GSI), gas II (GSII), and curtain gas (CUR) were maintained at 50, 55, and 35 psi, respectively, while the collision-activated dissociation (CAD) was set to medium. Instrument tuning and mass calibration were conducted using 10 and 100 μmol/L polypropylene glycol solutions in QQQ and LIT modes, respectively. QQQ scans were acquired as MRM experiments, with the collision gas (nitrogen) set to medium. The declustering potential (DP) and collision energy (CE) for individual MRM transitions were optimized further. A specific set of MRM transitions was monitored for each period in accordance with the metabolites eluted during that time.

### 4.4. Qualitative and Quantitative Analysis of Metabolites

Based on the self-built database GB-PLANT, qualitative analysis of substances was conducted using secondary spectral information. During the analysis, isotope signals, repeated signals containing K^+^, Na^+^, NH_4_^+^, and fragment ions representing larger molecular weight substances were removed. Metabolite mass spectrometry analysis data for various samples were obtained using Analyst 1.6.3. The peak areas of all mass spectral peaks of the substances were integrated, and the relative content of each component was calculated using the peak area normalization method. An internal standard was employed for data quality control (QC) to ensure reproducibility. Samples exhibiting metabolite features with a relative standard deviation (RSD) of QC greater than 30% were excluded from further analysis. The identified compounds were searched for classification and pathway information in the KEGG, HMDB, and LipidMaps databases. Based on the grouping information, difference multiples were calculated and compared, while a T-test was utilized to assess the significance of the differences, indicated by the p-value for each compound. The R language package ‘ropls’ was employed to perform OPLS-DA modeling, and 200 permutation tests were conducted to verify the reliability of the model. The Variable Importance in Projection (VIP) value of the model was calculated using multiple cross-validation. A combined approach incorporating the difference multiple, the *p*-value, and the VIP value of the OPLS-DA model was utilized to screen for differential metabolites. The screening criteria included a fold change (FC) greater than 1, a *p*-value less than 0.05, and a VIP value greater than 1. The significance of the differential metabolites in KEGG pathway enrichment was assessed using a hypergeometric distribution test.

### 4.5. RNA-Seq Library Preparation and Sequencing

The extraction of total RNA was performed according to the guidelines provided by the Plant RNA Extraction Kit (Sangon Biotechnology Company, https://www.sangon.com/, accessed on 20 September 2019; Shanghai, China). The quality of the extracted RNA was assessed through electrophoresis on a 1.5% agarose gel. mRNA was isolated from the total RNA using an mRNA capture beads kit, followed by the synthesis of double-stranded cDNA. Subsequent steps included terminal repair, the addition of a poly-A tail, and the incorporation of connectors. Library amplification products were purified using Hieff NGS™ DNA Selection Beads (Sangon Biotechnology Company, https://www.sangon.com/, accessed on 20 September 2019; Shanghai, China). Once the database was successfully constructed, sequencing was conducted using the Illumina HiSeq™ 2500 platform from the Shanghai Bioengineering Company.

The transcriptome sequence was provided in Excel format by Sangon Biotechnology Co., Ltd. (Shanghai, China) in 2019 and cannot be uploaded to a public repository. Due to the age of the sequence, the company has deleted the transcriptome sequence for this project (https://www.sangon.com/, accessed on 20 September 2019; Contract No.: MRNA192916QH). Instead, we summarized the transcript FPKM and GenBank Number of 38 Unigenes in our paper in Tables S6 and S8. 

### 4.6. Transcript Assembly and Analysis

The raw image data files obtained from Illumina Hiseq^TM^ are converted into raw sequencing sequences (Sequenced Reads). The results are stored in fastqc report, which includes summary table of raw data statistics, sample sequencing raw sequence data statistics, and FastQC visualization evaluation results. Clean data are assembled into a transcript using Trinity, with the parameter min_kmer_com2 set, while the other parameters remain at their default values. Annotations from CDD, KOG, COG, NR, NT, PFAM, Swissprot, and TrEMBL are obtained using NCBI Blast+. Gene Ontology (GO) functional annotation is derived based on the protein annotation results from Swissprot and TrEMBL, following the annotation information from Uniprot. The original data are filtered to yield effective clean data, which are then spliced and assembled into Unigene. Subsequently, Unigene is compared with a bioinformatics database to derive gene annotation results. The expression differences in Unigene are analyzed, and functions are predicted using the GO databases. Finally, the synthetic pathway of AsA is identified through GO gene enrichment analysis. Genes involved in AsA metabolism are compared at each stage of fruit development, with those exhibiting significant differences at each developmental stage selected for experimental verification. By comparing the transcriptome data from three developmental stages of *L. chinense* fruit, differentially expressed genes at each fruit stage are identified.

### 4.7. Gene Expression Analysis by RT-qPCR

Gene-specific primers were designed using Primer Premier 5 software and synthesized by Aoke Dingsheng Biotechnology Co., Ltd. (Beijing, China). The primers utilized in this study are listed in Table S4. Total RNA was extracted from *L. chinense* fruit using the FastPure^®^ Plant Total RNA Isolation Kit (suitable for polysaccharides and polyphenolics-rich samples). The integrity of the RNA was assessed using 1.2% agarose gel electrophoresis, while the RNA concentration was measured with a UV-visible spectrophotometer (BioSpec-nano, Shimadzu, Kyoto, Japan). The synthesis of the first strand of cDNA was performed using a reverse transcription kit (Hiscript^®^ RT-qPCR Supermax, Vazyme Biotech Co., Ltd., Nanjing, China), and the resulting reverse transcription products were stored at −20 °C for subsequent experiments.

Using the first strand cDNA synthesized above as the template, RT-qPCR was performed with the ChamQ Universal SYBR qPCR Master Mix kit. The RT-qPCR reaction system consisted of 10 μL of 2× ChamQ Universal SYBR qPCR Master Mix, 7.2 μL of ddH_2_O, 0.4 μL of upstream and downstream primers (Table S4), and 2 μL of cDNA, resulting in a total volume of 20 μL. The reaction procedure included denaturation at 95 °C for 30 s, followed by 40 cycles of a cyclic reaction at 95 °C for 5 s and 60 °C for 30 s, and concluded with a melting curve step at 95 °C for 15 s, 60 °C for 50 s, and 95 °C for 15 s. *L. chinense GAPDH* (Genbank Number: XM_060314892.1) was utilized as the reference gene and a negative control was established. Three replicates were conducted for each group, and the reactions were carried out on the QuantStudio 6 Flex PCR instrument (Applied Biosystems, Shanghai, China) [51].

### 4.8. Determination of Physiological Indices

The fresh weight of *L. chinense* fruits was measured using an electronic balance (1/10,000, ESJ200-4B, Shenyang, China). The dimensions of the fruits, including longitudinal length and radial width, were measured with a vernier caliper (DELIXI DWKC-2012, Hangzhou, China). The quantification of glutathione (GSH and GSSG) was conducted spectrometrically, following the methodology described by Lwalaba et al. [52]. GSH was oxidized and reduced by the addition of 5, 5′-dithio-bis (2-nitrobenzoic acid) (DTNB) and glutathione reductase in the presence of NADPH. The reduced glutathione (GSH) content was calculated as the difference between total glutathione and oxidized glutathione (GSSG). The contents of ascorbic acid (AsA), dehydroascorbic acid (DHA), GSSG, and GSH were determined using an enzyme-linked immunosorbent assay (ELISA) kit (Jiangsu Boshen Biotechnology Co., Ltd., Nanjing, China). The activities of GalDH, APX, AO, DHAR, MHDAR, and GR were assessed using the Biobox-AK from Box Biotechnology Co., Ltd. (Beijing, China).

### 4.9. Data Statistics and Analysis

Principal component analysis (PCA) of metabolites was conducted using the ‘prcomp’ function from R version 3.6.1. The ‘prcomp’ package facilitated the analysis, while ‘factoextra’ was employed for ggplot2-based visualization. In the PCA score chart, the abscissa represents the first principal component (PC1), and the ordinate represents the second principal component (PC2). The mean and standard deviation of the three biological replicates were calculated using QuantStudio™ Real-time PCR software 6. All data in this study were analyzed based on three independent biological replicates, including RNA sequencing and RT-qPCR analysis. Statistical analysis was performed using SPSS version 20 (IBM, Armonk, NY, USA), with results expressed as the mean ± SEM. One-way ANOVA followed by Dunnett’s post hoc test was utilized for statistical comparisons. Additionally, OmicShare tools (https://www.omicshare.com/, accessed on 10 June 2024) were employed to assess the correlation between the relative expression levels and metabolic compounds in the fruits of *L. chinense*.

## 5. Conclusions

In this study, we identified nine differential metabolites associated with AsA synthesis pathway in *L. chinense* fruits at three developmental stages. These metabolites included 1D-Myo-Inositol-1,4-BP, D-Fructose, L-(+)-Arabinose, I-Inositol, L-Arabinitol, D-Galactose-1-P, lactose, α-D-Glucose, and D-Glucose-6-P. Notably, the levels of D-Glucose-6-P, D-Galactose-1-P, and D-Fructose increased as *L. chinense* fruit developed. Concurrently, we observed an increase in fresh weight, longitudinal length, and radial width, while the concentrations of DHA, total AsA (AsA + DHA), and GSSG were decreased within the AsA-GSH cycle. GalDH and DHAR were enzymes contributing to the high accumulation of total AsA. Additionally, *PMM1*, *PMM5*, *GME2*, and *GME3* were recognized as key regulatory genes in the L-galactose pathway, which was crucial for AsA synthesis. *DHAR1* and *DHAR2* were identified as major positive regulatory genes for total AsA in the AsA-GSH cycle. These findings provide evidence for the regulatory mechanisms underlying AsA metabolism in *L. chinense* fruits and lay the foundation for the utilization of nutrients in *L. chinense* fruits.

## Figures and Tables

**Table 1:** Nine differential metabolites of AsA synthesis pathway in *L. chinense* fruits.

ID	Name	Class I	Class II	Ratio of Metabolite Contents	
				CCF/GF	RRF/GF
NEG_q4	1D-Myo-Inositol 1,4-BP	Sugars and alcohols	Other	0.94	1.42
NEG_q83	D-Fructose	Sugars and alcohols	Monosaccharides	7.66	12.38
NEG_q155	L-(+)-Arabinose	Sugars and alcohols	Monosaccharides	1.50	1.43
NEG_q137	I-Inositol	Organic acid	Other	1.48	1.11
NEG_q153	L-Arabinitol	Sugars and alcohols	Sugar Alcohols	0.60	0.21
NEG_q122	D-Galactose-1-P	Sugars and alcohols	Hexosephosphates	1.01	2.75
POS_q130	Lactose	Sugars and alcohols	Disaccharides	1.40	0.05
NEG_q54	Alpha-D-Glucose	Sugars and alcohols	Monosaccharides	0.81	1.20
NEG_q88	D-Glucose 6-P	Sugars and alcohols	Hexosephosphates	1.43	4.11

## Data Availability

In our study, GenBank Numbers of 38 unigenes were in Table S8.

## Supplementary Materials

The following supporting information can be downloaded at: https://www.mdpi.com/article/10.3390/ijms252111394/s1.

## Abbreviations

AO: ascorbate oxidase; APX, ascorbic acid peroxidase; AsA, ascorbic acid; DHA, dehydroascorbic acid; DHAR, dehydroascorbic acid reductase; GalLDH, L-Galactosate-1,4-lactone dehydrogenase; GalUR, D-Galacturonate reductase; GGP, GDP-l-Galactose phosphorylase; GLDH, L-Galactono-1,4-lactone dehydrogenase; GME, GDP-D-Mannose-3′,5′-epimerase; GMP, GDP-D-Mannose pyrophosphorylase; GPP, l-Galactose-1-P phosphatase; GR, glutathione reductase; GSH, reduced glutathione; GSSG, disulfide glutathione; GulLO, L-Gulono-1,4-lactone oxidase; MDHAR, monodehydroascorbic acid reductase; MIOX, myoinositol oxygenase; NADP, nicotinamide adenine dinucleotide phosphate; PGI, phosphoglucose isomerase; PMI, phosphomannose isomerase; PMM, phosphomannose mutasee.
