# Supplementary material for: Identification of Key Enzymes and Genes Modulating L-Ascorbic Acid Metabolism During Fruit Development of Lycium chinense by Integrating Metabolome, Transcriptome, and Physiological Analysis
Source: Int J Mol Sci. 2024 Oct 23;25(21):11394. doi: 10.3390/ijms252111394 (PMC11547089; doi:10.3390/ijms252111394)
Supplement: Supplementary file 1 [file ijms-25-11394-s001.zip › ijms-3219193-supplementary.pdf]

Table S1 The KEGG metabolites annotation of *L. chinense* fruits

First Category	Second Category	ko ID	DAM numbers
Amino acid metabolism	Arginine biosynthesis	ko00220	5
	Alanine, aspartate and glutamate metabolism	ko00250	6
	Glycine, serine and threonine metabolism	ko00260	8
	Cysteine and methionine metabolism	ko00270	9
	Valine, leucine and isoleucine degradation	ko00280	1
	Valine, leucine and isoleucine biosynthesis	ko00290	3
	Lysine biosynthesis	ko00300	3
	Lysine degradation	ko00310	7
	Arginine and proline metabolism	ko00330	4
	Histidine metabolism	ko00340	5
	Tyrosine metabolism	ko00350	4
	Phenylalanine metabolism	ko00360	7
	Tryptophan metabolism	ko00380	4
	Phenylalanine, tyrosine and tryptophan biosynthesis	ko00400	5
Biosynthesis of other secondary metabolites	Caffeine metabolism	ko00232	2
	Monobactam biosynthesis	ko00261	6
	Carbapenem biosynthesis	ko00332	2
	Neomycin, kanamycin and gentamicin biosynthesis	ko00524	6
	Indole alkaloid biosynthesis	ko00901	1
	Phenylpropanoid biosynthesis	ko00940	5
	Flavonoid biosynthesis	ko00941	7
	Isoflavonoid biosynthesis	ko00943	2
	Flavone and flavonol biosynthesis	ko00944	13

	Stilbenoid, diarylheptanoid and gingerol biosynthesis	ko00945	2
	Isoquinoline alkaloid biosynthesis	ko00950	1
	Tropane, piperidine and pyridine alkaloid biosynthesis	ko00960	5
	Betalain biosynthesis	ko00965	1
	Glucosinolate biosynthesis	ko00966	2
	Biosynthesis of various alkaloids	ko00996	5
	Biosynthesis of various plant secondary metabolites	ko00999	9
Carbohydrate metabolism	Glycolysis / Gluconeogenesis	ko00010	4
	Citrate cycle (TCA cycle)	ko00020	4
	Pentose phosphate pathway	ko00030	3
	Pentose and glucuronate interconversions	ko00040	8
	Fructose and mannose metabolism	ko00051	7
	Galactose metabolism	ko00052	11
	Ascorbate and aldarate metabolism	ko00053	5
	Starch and sucrose metabolism	ko00500	8
	Amino sugar and nucleotide sugar metabolism	ko00520	10
	Inositol phosphate metabolism	ko00562	3
	Pyruvate metabolism	ko00620	3
	Glyoxylate and dicarboxylate metabolism	ko00630	8
	Propanoate metabolism	ko00640	2
	Butanoate metabolism	ko00650	5
	C5-Branched dibasic acid metabolism	ko00660	4
Energy metabolism	Oxidative phosphorylation	ko00190	2
	Carbon fixation in photosynthetic organisms	ko00710	3
	Nitrogen metabolism	ko00910	1

	Sulfur metabolism	ko00920	5
Folding, sorting and degradation	Sulfur relay system	ko04122	1
Lipid metabolism	Fatty acid biosynthesis	ko00061	2
	Steroid biosynthesis	ko00100	2
	Glycerolipid metabolism	ko00561	4
	Glycerophospholipid metabolism	ko00564	5
	alpha-Linolenic acid metabolism	ko00592	2
	Sphingolipid metabolism	ko00600	2
	Biosynthesis of unsaturated fatty acids	ko01040	3
Membrane transport	ABC transporters	ko02010	27
Metabolism of cofactors and vitamins	Ubiquinone and other terpenoid-quinone biosynthesis	ko00130	2
	Thiamine metabolism	ko00730	4
	Riboflavin metabolism	ko00740	1
	Nicotinate and nicotinamide metabolism	ko00760	8
	Pantothenate and CoA biosynthesis	ko00770	4
	Biotin metabolism	ko00780	2
	Lipoic acid metabolism	ko00785	1
	Folate biosynthesis	ko00790	2
	Porphyrin metabolism	ko00860	3
Metabolism of other amino acids	beta-Alanine metabolism	ko00410	5
	Taurine and hypotaurine metabolism	ko00430	5
	Phosphonate and phosphinate metabolism	ko00440	2
	Cyanoamino acid metabolism	ko00460	4
	D-Amino acid metabolism	ko00470	14
	Glutathione metabolism	ko00480	2

Metabolism of terpenoids and polyketides	Polyketide sugar unit biosynthesis	ko00523	1
	Terpenoid backbone biosynthesis	ko00900	1
	Monoterpenoid biosynthesis	ko00902	2
	Carotenoid biosynthesis	ko00906	1
	Zeatin biosynthesis	ko00908	7
	Sesquiterpenoid and triterpenoid biosynthesis	ko00909	2
Nucleotide metabolism	Purine metabolism	ko00230	6
	Pyrimidine metabolism	ko00240	6
Signal transduction	Phosphatidylinositol signaling system	ko04070	2
Signal transduction	Plant hormone signal transduction	ko04075	6
Translation	Aminoacyl-tRNA biosynthesis	ko00970	9
Total			386

Table S2 The number of differentially metabolites in *L. chinense* fruits among three stages

group	GF vs RRF		GF vs CCF		CCF vs RRF	
	Up-regulated	Down-regulated	Up-regulated	Down-regulated	Up-regulated	Down-regulated
Alkaloids	13	24	9	26	13	18
Amino acids	27	24	21	24	26	16
Coumarins	2	4	2	4	4	3
Flavonoids	4	17	7	13	7	17
Ketones, Aldehydes, Acids	6	7	4	6	4	5
Lignans	1	4	0	4	0	2
Lipid	5	9	7	9	3	7
Nucleosides	0	1	0	0	0	1
Nucleotides	2	5	1	5	3	4
Organic acid	17	18	9	19	19	19
Phenylpropanoids	0	4	2	3	0	2
Polyphenols	9	12	7	14	9	12
Quinones	0	1	0	2	0	0
Sugars and alcohols	12	15	25	7	14	16
Steroids	1	2	1	2	1	3
Terpenoids	11	20	9	17	11	17
Vitamins	1	2	0	3	2	2
Xanthones	0	2	0	0	0	0
Others	10	19	8	13	12	17
Total	121	190	112	171	128	161

Table S3 The metabolites of Sugar and alcohol, Ketones, Aldehydes, Acids in *L. chinense* fruits among three stages

Number	Name	Class I	Class II
1	Alpha-D-Glucose	Sugars and alcohols	Monosaccharides
2	D-Arabinose	Sugars and alcohols	Monosaccharides
3	D-Fructose	Sugars and alcohols	Monosaccharides
4	D-(+)-Glucose	Sugars and alcohols	Monosaccharides
5	D-Glucose 6-Phosphate (Disodium Salt)	Sugars and alcohols	Monosaccharides
6	Dl-Xylose	Sugars and alcohols	Monosaccharides
7	D-Mannoheptulose	Sugars and alcohols	Monosaccharides
8	D-Tagatose	Sugars and alcohols	Monosaccharides
9	L-(+)-Arabinose	Sugars and alcohols	Monosaccharides
10	L(+)-Arabinose	Sugars and alcohols	Monosaccharides
11	L-Glucose	Sugars and alcohols	Monosaccharides
12	L-Xylose	Sugars and alcohols	Monosaccharides
13	(2S,3R,4S,5R)-2-Amino-3,4,5,6-Tetrahydroxyhexanal Hydrochloride	Sugars and alcohols	Monosaccharides
14	(3S,4R,5S)-1,3,4,5,6-Pentahydroxyhexan-2-One	Sugars and alcohols	Monosaccharides
15	D-Galactosamine Hcl	Sugars and alcohols	Monosaccharides
16	D-Arabitol	Sugars and alcohols	Sugar Alcohols
17	D-Mannitol	Sugars and alcohols	Sugar Alcohols
18	D-Sorbitol	Sugars and alcohols	Sugar Alcohols
19	Dulcite	Sugars and alcohols	Sugar Alcohols
20	L-Arabinitol	Sugars and alcohols	Sugar Alcohols
21	Ribitol	Sugars and alcohols	Sugar Alcohols
22	(-)-Vibo-Quercitol	Sugars and alcohols	Sugar Alcohols
23	D-(+)-Maltose Monohydrate	Sugars and alcohols	Disaccharides
24	Maltose	Sugars and alcohols	Disaccharides

25	Sucrose	Sugars and alcohols	Disaccharides
26	Turanose	Sugars and alcohols	Disaccharides
27	Lactose	Sugars and alcohols	Disaccharides
28	Lactulose	Sugars and alcohols	Disaccharides
29	Clorobiocin	Sugars and alcohols	Glycosides
30	Uridine 5'-Diphospho-D-Glucose	Sugars and alcohols	Glycosides
31	Acarbose	Sugars and alcohols	Glycosides
32	Kaempferol-3-O-B-D-Glucosyl(1-2)Rhamnoside	Sugars and alcohols	Glycosides
33	Keracyanin (Chloride)	Sugars and alcohols	Glycosides
34	Schizandriside	Sugars and alcohols	Glycosides
35	2'''-N-Acetyl-6'''-Deamino-6'''-Hydroxyparomomycin Ii	Sugars and alcohols	Polyol
36	3''-Oxogentamicin A2	Sugars and alcohols	Polyol
37	6'''-Deamino-6'''-Oxoparomomycin Ii	Sugars and alcohols	Polyol
38	Allitol	Sugars and alcohols	Polyol
39	D-(+)-Melezitose	Sugars and alcohols	Polysaccharides
40	Inulin	Sugars and alcohols	Polysaccharides
41	Maltopentaose	Sugars and alcohols	Polysaccharides
42	1F-Fructofuranosylnystose	Sugars and alcohols	Polysaccharides
43	D-Galacturonic Acid (Hydrate)	Sugars and alcohols	Sugar Acids
44	N-Acetylneuraminic Acid	Sugars and alcohols	Sugar Acids
45	8-Demethyl-8-(2-O-Methyl-Alpha-L-Rhamnosyl)Tetracenomycin C	Sugars and alcohols	Tertiary Alcohol
46	1D-Myo-Inositol 1,4-Bisphosphate	Sugars and alcohols	Other
47	1-Phenylethanol	Sugars and alcohols	Other
48	D(+)-Raffinose (Pentahydrate)	Sugars and alcohols	Other
49	Gluconate (Sodium)	Sugars and alcohols	Other

50	Sodium (3S,4R,5R)-6-((Hydrogenphosphonato)Oxy)-3,4,5-Trihydroxy-2-Oxohexyl Phosphate Octahydrate	Sugars and alcohols	Other
51	A-L-Rhamnose Monohydrate	Sugars and alcohols	Other
52	Capsidiol	Sugars and alcohols	Other
53	Dl-Panthenol	Sugars and alcohols	Other
54	Glucose 1-Phosphate	Sugars and alcohols	Other
55	Rutinose	Saccharides And Alcohols	Other
56	1,6-anhydro-β-D-glucose	Saccharides And Alcohols	Other
57	Verbascose	Saccharides And Alcohols	Other
58	Isomaltose	Sugars and alcohols	Glucans
59	Γ-Cyclodextrin	Sugars and alcohols	Glucans
60	D-Glucose 6-Phosphate	Sugars and alcohols	Hexosephosphates
61	Galactose 1-Phosphate	Sugars and alcohols	Hexosephosphates
62	N-Acetyl-D-Glucosamine	Sugars and alcohols	Amino Sugars
63	N-Acetyl-D-Mannosamine	Sugars and alcohols	Amino Sugars
64	Melezitose	Sugars and alcohols	Oligosaccharides
65	(R)-2-Hydroxy-2-Phenylacetamide	Sugars and alcohols	Aromatic Alcohol
66	C16 Sphingosine	Sugars and alcohols	Amino Alcohols
67	2-Phenylacetaldehyde	Ketones, Aldehydes, Acids	Other
68	4-Methoxybenzaldehyde	Ketones, Aldehydes, Acids	Benzaldehydes
69	Acetylpyrazine	Ketones, Aldehydes, Acids	Aromatic Ketone
70	Allantoic Acid	Ketones, Aldehydes, Acids	Other
71	Azelaic Acid	Ketones, Aldehydes, Acids	Carboxylic Acids
72	D-Fructose-6-Phosphate (Disodium) Salt	Ketones, Aldehydes, Acids	Sugar Acids
73	D-Gluconic Acid	Ketones, Aldehydes, Acids	Sugar Acids
74	Dl-Glyceric Acid	Ketones, Aldehydes, Acids	Sugar Acids

75	Hypotaurine	Ketones, Aldehydes, Acids	Other
76	Indolelactic Acid	Ketones, Aldehydes, Acids	Indole And Its Derivatives
77	Marilactone	Ketones, Aldehydes, Acids	Polyketides
78	Rubinaphthin A	Ketones, Aldehydes, Acids	Polyketides
79	(S)-3-(4-Hydroxyphenyl)-2-Hydroxypropionic Acid	Ketones, Aldehydes, Acids	Phenylpropanoic Acids
80	Sn-Glycerol 3-Phosphate	Ketones, Aldehydes, Acids	Other
81	Sulcatone	Ketones, Aldehydes, Acids	Methyl Ketone
82	Sulfinpyrazone	Ketones, Aldehydes, Acids	Other
83	Uridine 5'-Monophosphate Disodium Salt	Ketones, Aldehydes, Acids	Other
84	1-Phenylpentan-1-One	Ketones, Aldehydes, Acids	Aromatic Ketone
85	Benzoyleneurea	Ketones, Aldehydes, Acids	Other
86	Capillin	Ketones, Aldehydes, Acids	Other

Table S4. Overview of the RNA-seq data in *L. chinense* fruits from three stages.

	**Raw Reads**	**Clean Reads**	**Clean bases**	**Q20 Bases Ratio (%)**	**Q30 Bases Ratio (%)**	**GC Bases Ratio (%)**
GF1	44,710,504	43,630,536	6.04G	99.03%	96.09%	44.18%
GF2	47,008,126	45,934,846	6.42G	99.00%	96.00%	43.99%
GF3	47,156,284	45,914,848	6.36G	99.01%	96.05%	44.66%
CCF1	48,531,116	47,086,152	6.50G	98.85%	95.52%	43.81%
CCF2	45,780,196	44,237,330	5.98G	98.79%	95.41%	45.39%
CCF3	47,421,376	45,969,218	6.32G	98.85%	95.55%	45.11%
RRF1	44,212,860	43,110,618	6.02G	98.86%	95.55%	43.26%
RRF2	45,926,526	44,720,502	6.06G	98.88%	95.64%	43.25%
RRF3	42,916,958	41,822,904	5.74G	98.83%	95.49%	43.50%

Table S5 38 Unigenes in AsA metabolism of *L. chinense* fruits

Number	Name	Transcript No.	Pathway
1	PMI1	DN39029_c1_g4	
2	PMI2	DN39029_c1_g2	
3	PMM1	DN33963_c0_g1	
4	PMM2	DN34059_c6_g2	
5	PMM3	DN35722_c0_g2	
6	PMM4	DN37261_c1_g1	L-Galactose Pathway
7	PMM5	DN38020_c3_g3	
8	PMM6	DN38233_c0_g1	
9	GME1	DN34862_c8_g1	
10	GME2	DN38240_c2_g2	
11	GME3	DN38240_c2_g1	

12	GalDH	DN36619_c1_g3	
13	GLDH	DN38093_c0_g1	
14	GulLO1	DN35533_c0_g1	L-Gulose Pathway
15	GulLO2	DN36994_c2_g3	
16	MIOX1	DN36482_c0_g2	Myo-inositiol Pathway
17	MIOX2	DN36482_c0_g1	
18	MIOX3	DN36482_c2_g3	
19	MIOX4	DN36482_c2_g2	
20	AO1	DN27503_c0_g1	AsA-GSH Cycle
21	AO2	DN36414_c1_g3	
22	AO3	DN35962_c2_g2	
23	AO4	DN38228_c0_g1	
24	AO5	DN33578_c5_g2	
25	AO6	DN35462_c0_g4	
26	AO7	DN39233_c3_g2	
27	APX1	DN37079_c1_g1	
28	APX2	DN40486_c1_g6	
29	APX3	DN33398_c0_g5	
30	APX4	DN36154_c0_g2	
31	APX5	DN38645_c0_g2	
32	APX6	DN29606_c0_g1	
33	MDHAR1	DN35546_c0_g2	
34	MDHAR2	DN36216_c2_g1	
35	DHAR1	DN34151_c0_g1	
36	DHAR2	DN22410_c0_g1	

37	GR1	DN37505_c3_g1
38	GR2	DN36349_c0_g3

Table S6 FPKM of 38 Unigenes

	Name	#Gene	GF1	GF2	GF3	CCF1	CCF2	CCF3	RRF1	RRF2	RRF3
1	PMI1	TRINITY_DN39029_c1_g4	1.99323	0.64871	1.151	0.224459	0	0.464986	0	0.12202	0.261614
2	PMI2	TRINITY_DN39029_c1_g2	13.0789	5.82801	9.11536	6.54776	2.35174	3.98041	6.65387	4.983007	6.857172
3	PMM1	TRINITY_DN33963_c0_g1	17.10823	21.94548	30.95683	37.39031	28.82479	25.09287	51.26474	61.32746	58.48123
4	PMM2	TRINITY_DN34059_c6_g2	9.581605	8.847791	7.214104	6.114143	2.666448	2.744515	12.0534	11.34033	10.97912
5	PMM3	TRINITY_DN35722_c0_g2	9.998083	13.96747	15.81055	23.38904	30.93239	31.2956	10.72342	9.775833	7.912295
6	PMM4	TRINITY_DN37261_c1_g1	12.67335	12.62704	11.80171	15.2963	11.5433	11.15256	10.84113	16.09117	13.9966
7	PMM5	TRINITY_DN38020_c3_g3	3.879984	16.07415	3.226697	16.41357	3.516321	9.141206	150.0077	138.3633	146.0094
8	PMM6	TRINITY_DN38233_c0_g1	155.9475	114.3992	118.9775	71.81448	27.35263	38.11528	101.0218	91.97414	99.84639
9	GME1	TRINITY_DN34862_c8_g1	109.5293	144.0552	135.8978	393.4932	91.08495	172.2681	358.8208	334.4038	505.014
10	GME2	TRINITY_DN38240_c2_g2	27.33359	35.34148	25.90736	18.88605	13.24365	18.54943	40.135	51.7973	35.37226
11	GME3	TRINITY_DN38240_c2_g1	83.33747	89.48248	83.79181	180.4415	45.2909	76.92617	218.2181	235.7245	357.786
12	GalDH	TRINITY_DN36619_c1_g3	37.69381	39.93919	16.92624	2.12733	0.832283	1.693274	1.424015	1.66121	1.640936
13	GLDH	TRINITY_DN38093_c0_g1	0	0.116863	0	0	0	0.12548	0	0.7896	0.141178
14	GulLO1	TRINITY_DN35533_c0_g1	0	0.073916	0	0	0	0	0.086158	0.336586	0
15	GulLO2	TRINITY_DN36994_c2_g3	12.20746	5.44436	11.9444	41.88176	15.7476	18.85887	23.62641	18.32966	23.41503
16	MIOX1	TRINITY_DN36482_c0_g2	0.478089	0	1.34704	1.89687	1.19359	1.52593	0.527693	0.805236	0.429363
17	MIOX2	TRINITY_DN36482_c0_g1	22.41296	23.45142	22.29159	27.94093	14.27397	17.66137	37.67311	27.73479	34.68867
18	MIOX3	TRINITY_DN36482_c2_g3	1.494268	2.421847	1.767066	5.08088	2.246078	2.54754	5.896966	3.688289	7.37996
19	MIOX4	TRINITY_DN36482_c2_g2	2.816415	1.72181	1.85458	0.967637	0.512612	0.677626	0.768295	1.178567	0.586727
20	AO1	TRINITY_DN27503_c0_g1	7.29626	7.07351	7.07383	0.092972	0.336051	0.264252	0.309355	1.2203	0.540815
21	AO2	TRINITY_DN36414_c1_g3	8.257212	11.42214	7.106432	6.438732	4.455916	8.530322	45.26004	45.21164	43.43931
22	AO3	TRINITY_DN35962_c2_g2	4.42372	8.81252	1.78008	3.10646	0.497102	0.949611	2.6929	1.02901	2.11237
23	AO4	TRINITY_DN38228_c0_g1	0.106843	0.426558	0.60663	0.149899	0.191445	2.783651	0.755562	1.95595	0.589157
24	AO5	TRINITY_DN33578_c5_g2	0.202655	0.305431	0.177724	0.443216	0.437052	4.43479	0.446745	2.42444	0.46873

25	AO6	TRINITY_DN35462_c0_g4	0.377855	0.59491	0.435154	14.6224	9.90891	18.81898	7.4637	5.50517	6.26006
26	AO7	TRINITY_DN39233_c3_g2	18.97225	26.00426	29.40385	38.73305	32.07147	31.97021	45.3753	69.54205	35.7717
27	APX1	TRINITY_DN37079_c1_g1	187.3067	202.6307	170.1763	116.2375	135.6152	129.0616	147.6487	211.5588	126.0785
28	APX2	TRINITY_DN40486_c1_g6	28.62174	48.98338	55.28005	317.4388	64.55624	157.607	606.9468	528.794	592.2807
29	APX3	TRINITY_DN33398_c0_g5	4.038446	5.33786	3.813592	5.177129	1.414503	1.87808	4.582385	3.645995	7.024342
30	APX4	TRINITY_DN36154_c0_g2	71.74561	69.65191	64.66077	40.46472	14.71941	18.92548	74.07063	62.69376	83.70594
31	APX5	TRINITY_DN38645_c0_g2	18.36168	25.87016	14.6413	6.871431	1.992806	5.3109	68.346	55.5265	84.52912
32	APX6	TRINITY_DN29606_c0_g1	2.20318	3.54437	1.12672	0.60974	0.554907	0.259575	1.15527	0.509557	0.333905
33	MDHAR1	TRINITY_DN35546_c0_g2	66.25578	107.3453	49.34383	24.73338	20.75371	34.07893	161.6527	183.2842	173.9743
34	MDHAR2	TRINITY_DN36216_c2_g1	26.97268	25.39152	25.68606	8.789313	7.275539	10.13463	56.60727	61.93624	63.3985
35	DHAR1	TRINITY_DN34151_c0_g1	52.61588	63.54094	32.94015	42.6833	32.6783	24.95804	23.55203	16.05998	27.08633
36	DHAR2	TRINITY_DN22410_c0_g1	42.9174	39.3619	49.6522	37.14369	57.8203	71.03266	11.54917	9.19243	12.27341
37	GR1	TRINITY_DN37505_c3_g1	28.79533	33.53732	21.01066	44.48103	22.61108	24.0557	57.27452	58.40787	52.02503
38	GR2	TRINITY_DN36349_c0_g3	46.53847	62.33239	46.27756	78.00869	34.83208	39.21503	152.6528	183.8025	167.4728

Table S7 The primers used for RT-qPCR

Number	Gene name	Primer Sequence (5′–3′)<br>(Forward/Reverse)	GenBank No.	Product length(bp)
1	*GLDH*	F:GATGGATCCTCTCAACAAGGAG<br>R:CTCCAGCCTACTCGATATCCTTC	PQ464761	93
2	*AO1*	F:GTTCTCCTCCAAGCACAGATG<br>R:AGAGCAGCAAGAGCAGTCAAG	KP712033.1	130
3	*AO2*	F:AGGCAGGACTTACAGATTCAGG<br>R:TTGTAGGGTGTGAGTCCCTTCT	PQ464762	115
4	*AO6*	F:ACCTACTCTGTCCTCATCACAGC	PQ464766	116

Number	Gene name	Primer Sequence (5′–3′) (Forward/Reverse)	GenBank No.	Product length(bp)
		R:GAGTTGCTGTAGCGAAGTACACC		
5	*AO7*	F:GCCAACAGGGGATTCTGATA	PQ464767	128
		R:GAGTCCATTCCAGGTGAGAAGA		
6	*APX2*	F:TTATCTGGTGCTCACACTCTGG	PQ464768	141
		R:TAGCTGGAGGAGACCTTCTTTG		
7	*APX3*	F:AGTCAAGAGGGTTGAGGATGAG	PQ464769	140
		R:CTTACTTGAAGCGTTGGTGGTG		
8	*APX4*	F:GGAAGAGTTCACTCATGGTGCT	PQ464770	135
		R:GTGACCTCAACTGCAACAACAC		
9	*APX6*	F:TGCTGACCTCTATCAGCTTGC	PQ464772	147
		R:CTCAAGTGTGGTGGACCTTGT		
10	*MDHAR1*	F:CCTGCACTTAGCAAGGCATAC	PQ464773	110
		R:TCTGCATACCACTCAGGAAGC		
11	*DHAR2*	F:AATACCCAACTCCCTCTCTCGT	PQ464775	111
		R:CGGTACCATCACTAGCATCCTT		
12	*GR1*	F:GAGCAAATCGGGTGAAGAAG	PQ490811	120
		R:CAGATTGCAACCTTAGCTCCAG		
13	*GR2*	F:GATCCTCATACGGTGGATGTG	PQ464776	105
		R:ATACTCGCTTCCAGGAACGTC		
14	*GAPDH*	F:GCTGTTGCGAGCATGGTG	XM_060314892.1	112
		R:GACAGCCCCTCAAACTCGTC		
15	*PMI1*	F: GTAATGACAGTTCGCAAGGAT	PO490808	161
		R: AAGTATGCAGCTAAGACACCA		
16	*PMI2*	F: GGTACGTGCTGGCTTAACTCC	PQ464745	246

Number	Gene name	Primer Sequence (5′–3′) (Forward/Reverse)	GenBank No.	Product length(bp)
		R: GTCACTGTTCCCTTTCCCTCC		
17	*PMM3*	F: CTATCGGGTACTGGGTCTGCT<br>R: AAGCGTCCAACCTGCTACAAC	PQ464748	252
18	*PMM4*	F: GCTGCCTCATCGTTCACTACAA<br>R: TTGCTTCGGTAACTGGCTCTG	PQ464749	142
19	*GME3*	F: GTCGTTTGAAGAGCGAGGGTC<br>R:CAGCAGCGAGGTTGAAGACAT	PQ464754	168
20	*GalDH*	F: GTCCCTCCAGGCACAGTTGAT<br>R:TCACTCCGACACCCTTGCTCT	PQ464755	109
21	*GulLO1*	F: GAATCCACAGCCATACTCCC<br>R:AACGACCACTGAATCTTCTCCT	PQ464759	180
22	*GulLO2*	F: GACATCACATATTACCGAAGC<br>R:ATAACCCTAATTGGTCGTAGC	PQ464760	211
23	*MIOX3*	F: ATGACGTGGTTGATGATAGTGA<br>R:CAACAACAGCCCATTGAGGAA	PQ464757	189
24	*MIOX4*	F: ACCCTGTTGGCTGTGCTTTCG<br>R:AGCTGATGGCAATGTTGTCCC	PQ464758	203

Table S8. Transcript No. and GenBank No. of 38 Unigenes

Order	Gene Name	Transcript No. (Unigene)	GenBank No.	Note
1	PMI1	TRINITY_DN39029_c1_g4	PO490808	Partial CDS
2	PMI2	TRINITY_DN39029_c1_g2	PQ464745	Complete CDS
3	PMM1	TRINITY_DN33963_c0_g1	PQ464746	Complete CDS

4	PMM2	TRINITY_DN34059_c6_g2	PQ464747	Complete CDS
5	PMM3	TRINITY_DN35722_c0_g2	PQ464748	Complete CDS
6	PMM4	TRINITY_DN37261_c1_g1	PQ464749	Complete CDS
7	PMM5	TRINITY_DN38020_c3_g3	PQ464750	Complete CDS
8	PMM6	TRINITY_DN38233_c0_g1	PQ464751	Complete CDS
9	GME1	TRINITY_DN34862_c8_g1	PQ464752	Complete CDS
10	GME2	TRINITY_DN38240_c2_g2	PQ464753	Complete CDS
11	GME3	TRINITY_DN38240_c2_g1	PQ464754	Complete CDS
12	GalDH	TRINITY_DN36619_c1_g3	PQ464755	Complete CDS
13	GLDH	TRINITY_DN38093_c0_g1	PQ464761	Complete CDS
14	GulLO1	TRINITY_DN35533_c0_g1	PQ464759	Complete CDS
15	GulLO2	TRINITY_DN36994_c2_g3	PQ464760	Complete CDS
16	MIOX1	TRINITY_DN36482_c0_g2	PQ490809	Partial CDS
17	MIOX2	TRINITY_DN36482_c0_g1	PQ464756	Complete CDS
18	MIOX3	TRINITY_DN36482_c2_g3	PQ464757	Complete CDS
19	MIOX4	TRINITY_DN36482_c2_g2	PQ464758	Complete CDS
20	AO1	TRINITY_DN27503_c0_g1	KP712033.1	Complete CDS
21	AO2	TRINITY_DN36414_c1_g3	PQ464762	Complete CDS
22	AO3	TRINITY_DN35962_c2_g2	PQ464763	Complete CDS
23	AO4	TRINITY_DN38228_c0_g1	PQ464764	Complete CDS
24	AO5	TRINITY_DN33578_c5_g2	PQ464765	Complete CDS
25	AO6	TRINITY_DN35462_c0_g4	PQ464766	Complete CDS
26	AO7	TRINITY_DN39233_c3_g2	PQ464767	Complete CDS
27	APX1	TRINITY_DN37079_c1_g1	KX981601.1	Complete CDS
28	APX2	TRINITY_DN40486_c1_g6	PQ464768	Complete CDS
29	APX3	TRINITY_DN33398_c0_g5	PQ464769	Complete CDS

30	APX4	TRINITY_DN36154_c0_g2	PQ464770	Complete CDS
31	APX5	TRINITY_DN38645_c0_g2	PQ464771	Complete CDS
32	APX6	TRINITY_DN29606_c0_g1	PQ464772	Complete CDS
33	MDHAR1	TRINITY_DN35546_c0_g2	PQ464773	Complete CDS
34	MDHAR2	TRINITY_DN36216_c2_g1	PQ464774	Complete CDS
35	DHAR1	TRINITY_DN34151_c0_g1	PQ490810	Partial CDS
36	DHAR2	TRINITY_DN22410_c0_g1	PQ464775	Complete CDS
37	GR1	TRINITY_DN37505_c3_g1	PQ490811	Partial CDS
38	GR2	TRINITY_DN36349_c0_g3	PQ464776	Complete CDS
